# Binding of Targeted Semiconducting Photothermal Polymer Nanoparticles for Intraperitoneal Detection and Treatment of Colorectal Cancer

**DOI:** 10.7150/ntno.29522

**Published:** 2020-03-26

**Authors:** Eleanor McCabe-Lankford, Bryce McCarthy, Margarita Arakelyan- Peters Berwick, Kiarash Salafian, Laura Galarza-Paez, Santu Sarkar, John Sloop, George Donati, April J. Brown, Nicole Levi-Polyachenko

**Affiliations:** 1 Department of Plastic and Reconstructive Surgery, Wake Forest School of Medicine, Medical Center Boulevard, Winston-Salem, NC 27157, USA.; 2 Department of Chemistry, Wake Forest University, Winston-Salem, NC 27109, USA.

**Keywords:** Hybrid Donor-Acceptor Polymer Particles, HDAPPS, disseminated colorectal cancer, nanoparticles

## Abstract

Nanoparticles offer many promising advantages for improving current surgical regimens through their ability to detect and treat disseminated colorectal cancer (CRC). Hybrid Donor-Acceptor Polymer Particles (HDAPPs) have recently been shown to fluorescently detect and thermally ablate tumors in a murine model. Here, HDAPPS were functionalized with hyaluronic acid (HA) to improve their binding specificity to CT26 mouse CRC cells using HA to target the cancer stem cell marker CD44. In this work, we compared the binding of HA functionalized HDAPPs (HA-HDAPPs) in *in vitro*, *ex vivo*, and *in vivo* environments. The HA-HDAPPs bound to CT26 cells 2-fold more *in vitro* and 2.3-fold higher than un-functionalized HDAPPs *ex vivo*. Compared to intraoperative abdominal perfusion, intraperitoneal injection prior to laser stimulation for nanoparticle heat generation provides a superior modality of HA-HDAPPs delivery for CRC tumor selectivity. Photothermal treatment of disseminated CRC showed that only HA-HDAPPs delivered via intraperitoneal injection had a reduction in the tumor burden, and these nanoparticles also remained in the abdomen following resolution of the tumor. The results of this work confirm that HA-HDAPPs selectively bind to disseminated CRC, with *ex vivo* tumors having bound HA-HDAPPs capable of photothermal ablation. HA-HDAPPs demonstrated superior binding to tumor regions compared to HDAPPs. Overall, this study displays the theranostic potential of HDAPPs, emphasizing their capacity to detect and photothermally treat disseminated CRC tumors.

## Introduction

Peritoneal carcinomatosis (PC) is a condition where many small tumors have disseminated within the peritoneal cavity. The presence of PC associated with CRC correlates with late-stage disease progression and very poor prognoses 1. Patients that present with PC have limited success from systemic chemotherapy and complete surgical resection of all of the micro-metastatic lesions is unlikely 2. Preoperative imaging using magnetic resonance imaging, computerized tomography, and positron emission tomography are not sensitive enough to detect small tumors (< 7 mm) and tumors smaller than 5 mm are not palpable or visible to surgeons during cytoreductive surgery 3,4. Studies have shown that hyperthermic intraperitoneal (IP) chemotherapy (HIPEC) following cytoreduction can improve survival outcomes, but this treatment only penetrates a few millimeters into tissue, which may leave behind smaller lesions that may also be resistant to chemotherapy, thereby instigating recurrence 5,6. The utilization of nanomaterials as adjuvants for identifying and eliminating microscopic disseminated tumors provides a promising opportunity to reduce residual disease, a key aspect to improving survival.

Currently, numerous nanoparticle types and formulations have been explored as adjuvants for targeting and treating tumors *in vivo* 7-11. Polymer-based nanoparticles offer versatility in their tunable optical properties, easy synthesis, stability, near-infrared fluorescence, and photothermal capabilities 9,10,12-17. Hybrid Donor-Acceptor Polymer Particles (HDAPPs) are theranostic nanoparticles derived from a specific blend of two unique conjugated polymers: the donor-acceptor polymer poly[4,4-bis(2-ethylhexyl)-cyclopenta[2,1-b;3,4-b']dithiophene-2,6-diyl-alt-2,1,3-benzoselenadiazole-4,7-diyl] (PCPDTBSe), which has been synthesized to produce heat generating nanoparticles for photothermal ablation of tumors, and the fluorescent conjugated polymer poly[(9,9-dihexylfluorene)-co-2,1,3-benzothiadiazole-co-4,7-di(thiophen-2-yl)-2,1,3-benzothiadiazole] (PFBTDBT10) 10,12,14. The PCPDTBSe and PFBTDBT10 blend used to yield HDAPPs has been shown to produce on-demand heat generation and a unique far-red fluorescence signal (825 nm) due to an amplified energy transfer phenomenon 10,18,19.

Non-targeted HDAPPs were previously used to photothermally ablate breast tumors using near-infrared (NIR) laser irradiation to stimulate the nanoparticles, plus they were easily detected using fluorescence imaging 10. HDAPPs for the current study were functionalized with hyaluronic acid in order to target the cancer stem cell marker CD44. Both the standard and variant isoforms (CD44s and CD44v) have been shown to be expressed in primary CRCs and disseminated tumors from PC 20,21. CD44 is believed to aid in the formation of PC due to its role as an adhesion molecule by binding to the ECM protein hyaluronan.^20-24^ Hyaluronic acid (HA) is a natural polysaccharide composed of repeating disaccharide units of (1-3)-β linked *N*-acetyl-D-glucosamine and (1-4)-β linked D-glucuronic acid and has previously been coupled to nanoparticles for targeted cancer treatment 9,25-28.

For this study, the binding of HA-functionalized H-DAPPs (HA-HDAPPs) and non-functionalized HDAPPs to CRC cells and tumors were compared *in vitro*, *ex vivo*, and *in vivo.* The immunocompetent mouse line BALB/c, with its syngeneic undifferentiated colon carcinoma cell line (CT26), was used to induce a disseminated CRC model with peritoneal surface disease. Since disseminated abdominal cancers display high tumor numbers and tend to be poorly vascularized, HDAPPs were administered *in vivo* via an intraperitoneal injection or open abdominal perfusion as previously reported 29,30. The aim of this work was to describe the functionalization, binding capabilities, and dynamic theranostic properties of HDAPPs for future disseminated cancer detection and treatment.

## Materials and Methods

All chemicals were purchased from Sigma-Aldrich unless otherwise stated.

### Nanoparticle Synthesis and Characterization

Hybrid Donor-Acceptor Polymer Particles (HDAPPs) nanoparticles were synthesized according to previously published methods 10. The donor-acceptor polymer poly[4,4-bis(2-ethylhexyl)cyclopenta[2,1-b;3,4-b']dithiophene-2,6-diyl-alt-2,1,3-benzoselenadiazole-4,7-diyl] (PCPDTBSe) was combined with poly[(9,9-dihexylfluorene)-co-2,1,3-benzothiadiazole-co-4,7-di(thiophen-2-yl)-2,1,3-benzothiadiazole] (PFBTDBT10) at a 95% to 5% by weight ratio in tetrahydrofuran (polymer-mix). A 1 mL volume of 2 mg/mL polymer mix was added to 8 mL of 1 mg/mL aqueous pluronic F127 solution under constant horn sonication with a Branson Digital Sonifier with a microtip (1 minute, 20% amplitude). Nanoparticles were autoclaved (30 minutes, 121°C) then isolated by centrifugation. Large aggregates were removed by centrifugation of 5,500 rcf for 30 minutes and the subsequent supernatant was centrifuged at 16,800 rcf for 4 hours, to collect the nanoparticles. Nanoparticles composed of 100% PCPDTBSe (BSe NPs) were also prepared according to the same methodology. Photothermal conversion efficiencies (PCE) of HA-HDAPPs, HDAPPs and BSe NPs were determined, as described in the Supplementary Information.

### Hyaluronic-Acid-Coated Nanoparticles

Sterilized HDAPPs were coated with sterile chitosan (190 - 310 kDa) by vigorous stirring for 1 hour at room temperature (5 mL of 3 mg/mL chitosan in 2% acetic acid to 1 mL of 2 mg/mL of HDAPPs). The chitosan coating was used in order to bridge the addition of hyaluronic acid to the outer surface of the HDAPPs via an electrostatic interaction. Chitosan-coated HDAPPs were diluted in 15 mL of sterile 2% acetic acid and centrifuged for 2 hours at 8,600 rcf and then the pellets were re-suspended in 15 mL sterile water and centrifuged for an additional 2 hours at 8,600 rcf. The washed pellet was then added to 5 mL of sterile aqueous 100 µg/mL 66-99 kDa sodium hyaluronate (Lifecore Biomedical, MN) solution and subjected to 1 hour of vigorous stirring at room temperature in ambient light. The hyaluronic acid coated HDAPPs (HA-HDAPPs) were diluted into 10 mL of sterile water and centrifuged for 2 hours at 5,500 rcf to remove excess hyaluronic acid. All materials were stored at 4°C in the dark until use. Size was determined by dynamic light scattering (DLS) and transmission electron microscopy (TEM). The zeta potential was determined using a Zetasizer (Malvern) and a heat curve was generated by irradiating various concentrations of 200 µL of nanoparticles in a 96-well plate with 3.8 W/cm^2^ of 800 nm light. To evaluate the interaction of the various coatings on the nanoparticles solutions of chitosan, HA, HDAPPs, chitosan-coated HDAPPs, and HA-HDAPPs were lyophilized into a dry powder and evaluated for their respective Fourier transform infrared spectra (FTIR) using an Agilent Technologies, Cary 630 FTIR spectrometer.

### Cells

CT26.WT-Fluc-Neo (CT26) cells, a polyclonal population of the CT26.WT ATCC® CRL-2638™ mouse colorectal carcinoma line, transduced with lentivirus encoded with firefly luciferase and a neomycin resistance gene, were purchased from Imanis Life Sciences 2. Cells were grown in DMEM media supplemented with 10% FBS, 1X Penicillin/Streptomycin, and 0.4 mg/mL G418 in T225 flasks until reaching 80% confluency. Cells were trypsinized with 0.25% Trypsin until the cells lifted, and the Trypsin was neutralized with culture media. For *in vivo* injections, once cells were trypsinized and neutralized, they were pelleted, washed once with 1× PBS, and re-suspended in 1x PBS for injection.

### Nanoparticle Cytotoxicity MTS Assay

CT26 cells were plated (7,500 cells/well) in a 96-well plate and grown in culture media overnight. The next day, the media was removed and cells were rinsed once with sterile 1× PBS and plated with 100 µL of various concentrations (0, 10, 20, 40, 80, 160 µg/mL) of HA-HDAPPs in culture media in triplicate. The solutions were incubated with the cells for 24 hours. Afterwards, the nanoparticle solutions were removed; the cells were rinsed once with 1× PBS, and 120 µL of Celltiter 96 Aqueous non-radioactive cell proliferation assay solution (in media) was added to each well. The absorbance of the solutions was read at 490 nm and the background was subtracted. Cell survival was normalized to the 0 µg/mL HA-HDAPPs control.

### *In vitro* Nanoparticle Binding

CT26 cells were plated in a 48 well plate and grown to 80% confluency. The media was removed and the cells were rinsed once with saline. Nanoparticles (80 µg/mL of HDAPPs or HA-HDAPPs, or saline-based on our previous studies using HDAPPs for photothermal ablation.) were suspended in 0.9% saline and incubated on the cells for 30 minutes at 37°C (saline was used instead of PBS in order to mimic the *in vivo* binding perfusate). The solution was then removed, wells were rinsed once with sterile saline, and fresh saline was placed into the wells. The amount of nanomaterial that bound to cells was determined by measuring the Average Radiant Efficiency (ARE) ([photons/s/cm²/steradian] / [µW/cm²] and Total [photons/s] / [µW/cm²]) of the nanoparticle fluorescence signal excited at 465 nm with the indocyanine green (ICG) filter (810-875 nm) using the Living Image Software (PerkinElmer). An additional binding assay was also performed, using concentrations of 0.1, 0.5, 1, 1.75, 2.5, and 5 mg/ml of HDAPPs and HA-HDAPPs on 6000 adherent CT26 cells for 1hr at 4°C, via fluorescence evaluation using a plate-reader.

### *In vitro* Photothermal Ablation

Six thousand cells per seeded per well in a 96-well plate coated with poly-L-lysine. Cells were then exposed to 2.219 mg/ mL (based on the results from the binding assays determined using the fluorescence) of HDAPPs or PCPDTBSe (with and without HA coating), or media only for 1 hr at 4°C. Following treatment, wash cells twice with HEPES-buffered Saline (250 μM) containing 0.1% BSA. Fluorbrite transparent media was added and the wells were treated with 180 J/cm^2^, or no laser treatment. Twenty-four hours following treatment, cells were stained with trypan blue and counted using the Countess automatic cell counter.

### Animals

All animal studies were conducted in the Animal Research Core Facility at Wake Forest School of Medicine in accordance with institutional guidelines. Surgical procedures were approved by the Institutional Animal Care and Use Committee of Wake Forest University Health Sciences. Female BALB/c mice (5-7 weeks) were purchased from Charles River Laboratories, and sustained in a vivarium with a 12-hour light/dark schedule. Humane endpoints were determined either by disease burden (bioluminescence signal saturation) using IVIS, dramatic changes in weight or abdominal circumference, or animals showing signs of lethargy or distress.

### Development of the Disseminated Colorectal Cancer Model

The disseminated colorectal cancer model was performed in accordance with a previously published method 30. Six-week-old female BALB/c mice were anesthetized with 2% continuous vaporized isoflurane and injected intraperitoneally with 500 µL of 3× 10^6^ CT26.WT.Fluc.Neo (CT26, passage 3-10) cells suspended in 1× PBS. Cells were injected slowly while the needle was angled around the abdominal cavity. The mice were placed in supine position and their abdomens massaged for several minutes and the mice were kept under anesthesia for an additional five minutes in this position to allow the cells to settle to the dorsal side. Disease progression was monitored by measuring animal weight, abdominal girth, and tumor luminescence using IVIS.

### *Ex vivo* Nanoparticle Binding

A BALB/c mouse was given a 500 µL intraperitoneal injection of 3×10^6^ CT26 cells and the resulting tumors were excised 16 days later. The excised tumors were placed in saline, cut into equal sizes (~ 19 mg) and placed in a 96-well plate. The excised tumors were statically incubated for 30 minutes with either 100 µL of 0.9% saline, 80 µg/mL HDAPPs, or HA-HDAPPs at 37°C in a 5% CO_2_ humidified incubator. Afterwards, the incubating solutions were removed and the tumors were rinsed once with saline to remove unbound material, and replenished with 100 µL of fresh saline.

The plate was imaged with IVIS using 465 nm excitation and fluorescence emission was observed using the ICG filter. The signal of the nanoparticles bound to the tumors was normalized to control wells containing 100 µL of solutions of only 0.9% saline, 80 µg/mL HDAPPs, or HA-HDAPPs. The region of interest (ROI) of each well was determined using the Living Image software and the ARE signal compared to the controls. The total fluorescence signal in the saline only tumors was subtracted directly from the fluorescence signal in the nanoparticle incubated tumors and compared via a Student's t-test (Unequal Variances, p<0.04).

### Nanoparticle Delivery

Once the disseminated tumors were established, mice underwent an open abdominal perfusion as previously described 30. This technique is designed to mimic the peritoneal perfusion of chemotherapy that is delivered to human CRC patients during the HIPEC procedure. To develop this method, mice that presented with significant bioluminescence signals, indicative of disseminated disease, were anesthetized with 2% isoflurane (2 liters/min) then moved to the heated operative platform and kept on isoflurane inhalation at 1.5 liters/min. Mice were subcutaneously injected with 800 µL of Lactated Ringers Solution and had one drop of Rugby Artificial Tears Ointment added to each eye. A depilatory cream was used to remove the fur on the abdomen and the skin was disinfected with 7.5% betadine solution. The mouse was secured to a dry surgical towel and draped with Glad® Press'n Seal®. A 20 mm midline incision was made between the base of the sternum to the tail and through the peritoneal sac. The skin and peritoneum were secured to a metal ring with four 4-0 vicryl sutures to form the open “coliseum” and Rugby Artificial Tears Ointment was applied to the edges of the skin to prevent drying. Visible mucus, ascites, and disseminated surface tumors were removed with sterile gauze pads or pickups. Tumors (>2 mm) present on the peritoneum were removed with bipolar cauterizers.

The circuit used for the perfusion was comprised of a single inflow and outflow line attached to a Masterflex pump. The inflow line was placed in the abdomen, below the sternum, and the outflow line was placed against the lower abdominal cavity wall on the left side of the mouse. Approximately 6-8 mL of 80 µg/mL HA-HDAPPs (480-640 µg total) or saline, warmed to 37^o^C, was introduced into the circuit and perfused throughout the abdominal cavity for 30 minutes, with organ manipulation using a blunted probe. The flow in the circuit was set between 50-200 mL/hr and at the end of the perfusion, the abdomen was drained and the perfusate was collected. The abdomens were flushed three times with saline to remove unbound nanoparticle material. The sutures forming the coliseum were removed and the peritoneum and skin were closed with suture.

Alternatively, mice were anesthetized with isoflurane and intraperitoneally injected 24 hours prior to surgery with 1 mL of 100 µg/ mL of HDAPPs or HA-HDAPPs in saline. The IP injection required significantly less nanomaterial (100 µg of total material as opposed to the 480-640 µg needed in the perfusion circuit described above). This is one benefit of delivering the nanoparticles via IP injection compared to IP perfusion. In addition, our previous *in vitro* research has demonstrated that there is no significant difference between using 80 or 100 μg/ mL of HDAPPs for photothermal ablation and up to 200 μg of total material was safe for systemic administration 10. The next day, the mice were imaged by IVIS again to evaluate disease progression and nanoparticle localization. Mice were prepped for surgery as described above and had mucus, ascites, and tumors removed with gauze pads and bipolar cauterizers. The open abdomens were rinsed with saline three times while moving the organs with blunted probes in order to remove unbound nanoparticles. A heated saline perfusion was performed for 5 minutes to remove unbound nanoparticles and then the abdomens were closed using sutures.

### *Ex vivo* Nanoparticle Fluorescence and Disease Bioluminescence Detection

Mice that had developed disseminated tumors and undergone surgery were survived until a humane endpoint was reached. After mice were euthanized, they were quickly dissected and the organs were placed in separate, sterile, non-coated petri dishes and placed on ice. The tissues were imaged by IVIS for nanoparticle fluorescence then several drops of 150 µg/mL D-luciferin solution was added to the tissues and they were imaged for bioluminescence for 1s and for 10 s to observe the nanoparticle fluorescence. The fluorescence and bioluminescence images were overlaid to determine co-localization of the nanoparticles bound to the tumors and organs.

### *In vivo* Photothermal Ablation

A pilot study consisting of 3 mice per group (perfusion saline or HA-HDAPPs delivered via perfusion or IP injection, with or without laser stimulation) were used to evaluate photothermal efficacy *in vivo*. After filling the open abdomen with a light scattering agent (0.1% Nutrilipid 20% soybean oil emulsion, treatment mice had 180 J/ cm^2^ of 800nm light delivered one time. The laser beam diameter was expanded to 5 cm to cover the entirety of the abdomen. Total treatment time was 60s and the abdominal organs and solution were manipulated using non-metallic blunt probes during exposure.

Mice were weaned from the isoflurane and, once awake and stable, were given 0.05 mg/kg buprenorphine IP for pain. Disease progression and nanoparticle localization in the mice were measured by IVIS using luciferin injections for bioluminescence and the fluorescence of the nanoparticles was detected by exciting at 465 nm for 10s using an Indocyanine green ICG filter. Humane endpoints necessitating euthanasia were employed when the tumor burden caused bioluminescence saturation with 1 s excitation, or if there was evidence of intra-abdominal blood pooling, or mice became lethargic.

## Results

### Properties of Hyaluronic Acid Coated HDAPPs

HDAPPs were functionalized with HA to form HA-HDAPPs via an electrostatic interaction by first coating the HDAPPs with chitosan, a cationic biocompatible polysaccharide. Chitosan and HA assemblages, especially in nanocomplexes, have been cited in the literature for targeting of CD44 due to the facile targeting process and low toxicity of chitosan 27,33. CD44 was confirmed in CT26 cells using Western blotting and immunofluorescence (Supplementary Figure 1). The degree of glycosylation of CD44 regulates the receptor's binding capacity for HA. The most active form of CD44 is one that has the least glycosylation and thus can actively bind HA 26. HA constructs that are less than 20 kDa have been shown to have low binding affinity with CD44, but isoforms of HA closer to 100 kDa in size form tight, non-reversible bonds 28,34,35. Since nanoparticle size is an important factor for binding and colloidal stability, the functionalized HDAPPs needed to be as small as possible and the HA to be used for functionalization needed to be larger than 20 kDa. The HA isoform chosen for coating the HDAPPs in this study was a heterogeneous mixture ranging from 66-99 kDa.

**Figure 1:**
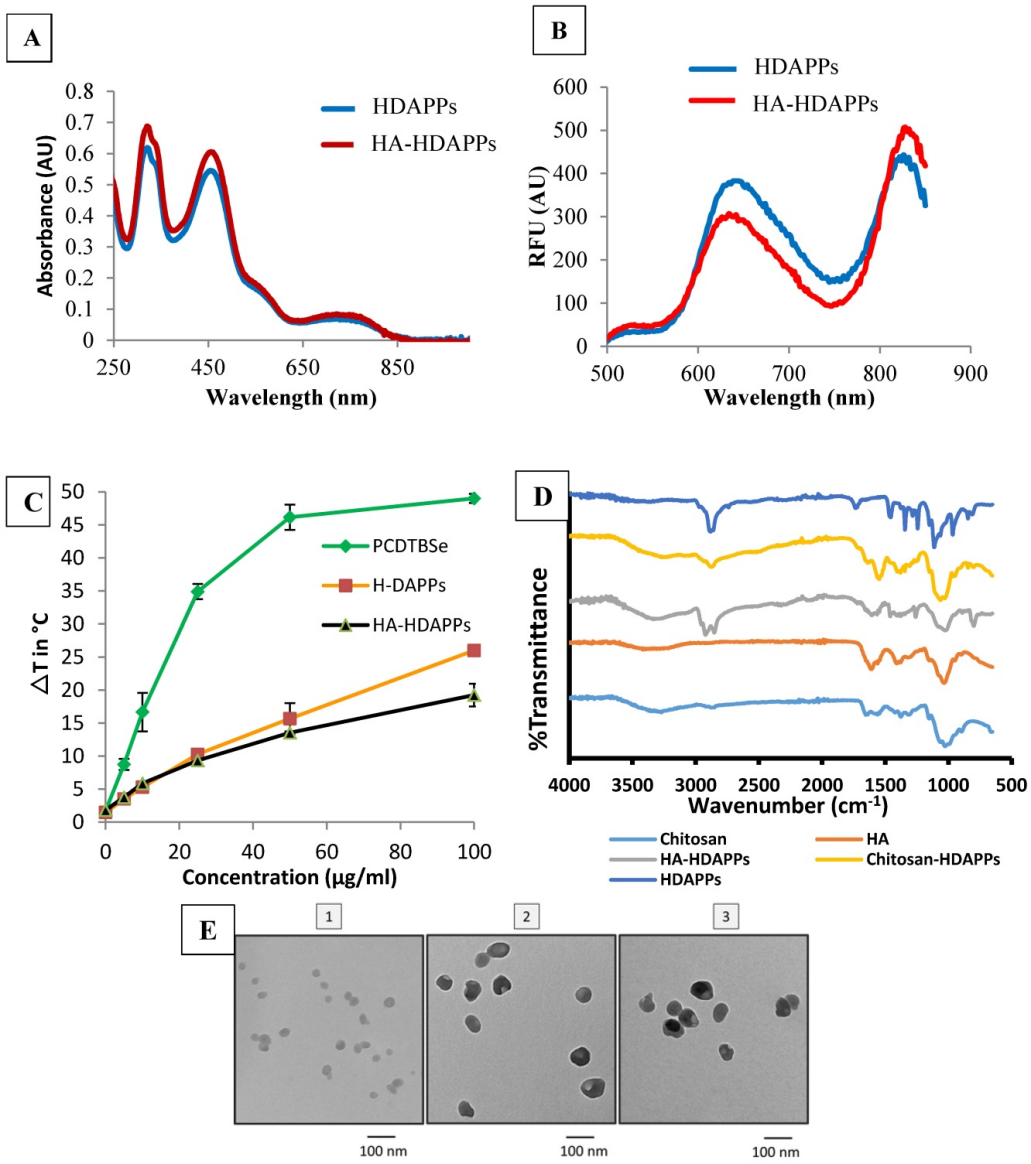
Nanoparticle Characterization** (A)** UV-Vis absorbance spectra of HDAPPs (blue) and HA-HDAPPs (red) (~14 ug/mL). (**B**) Fluorescence spectra of HDAPPs (blue) and HA-HDAPPs (red) (~14 ug/mL) excited at 465 nm. (**C**) Heating curve of HDAPPs and HA-HDAPPs irradiated with 3.8 W/cm^2^ of 800 nm light for 60 s. (**D**) FTIR spectroscopy of chitosan, HA, HDAPPs with no coating, HA, or chitosan coating. (**E**) TEM images of (1) HDAPPs, (2) Chitosan coated HDAPPs, and (3) Hyaluronic acid coated HDAPPs (HA-HDAPPs).

The HA-HDAPPs were found to have very similar optical properties to the non-coated HDAPPs (Figure 1). There was no shift in the UV-Vis absorbance spectra of the nanoparticles and only a slight amount of fluorescence signal dampening from H-DAPPs to HA-HDAPPs (Figure 1A&B). The HA-HDAPPs were found to achieve comparable heat-generation capacities as the non-functionalized HDAPPs using the same laser parameters (Figure 1C), although HA-HDPPs have about a 5°C lower ΔT. Also included in Figure 1C is the temperature generation of nanoparticles composed of 100% PCPDTBSe. These nanoparticles generate significantly higher temperatures compared to HDAPPs; however, they lack the fluorescent polymer. FTIR spectra of bare HDAPPs, or those coated with chitosan or HA, along with the chitosan and HA alone controls, are shown in Figure 1D. The width of the peak near 1100 cm^-1^ for the coated samples is representative of the addition of chitosan and/or HA. Functionalization of the HDAPPs is further supported by the presence of the peaks near 1600 and the newly observed peak near 2900, which is typically observed in both HA and chitosan. Transmission electron microscopy demonstrated that the addition of the coatings did not change the spherical shapes of the nanoparticles (Figure 1E). The hydrodynamic diameters of the nanoparticles increased with the chitosan (~123 nm) and hyaluronic acid coatings (~207 nm), and the zeta potentials shifted from negative, to positive with chitosan, then back to negative after the addition of the hyaluronic acid (Table 1). All nanoparticles remained stable in saline for weeks and the HA-coated versions were stable in saline for several days.

Photothermal conversion efficiency of HA-HDAPPs, HDAPPs, PCPDTBSe, gold nanoshells (NS) and gold nanorods (NR) was determined using the data presented in Supplementary Figure 2. The calculated photothermal conversion efficiencies were 57.09% for HDAPPs and 51.20% for HA-HDAPPs compared to 21.11% for AuNS, 62.95% for AuNR, and 62.4% for 100% PCPDTBSe nanoparticles. This suggests that HDAPPs are characteristically more efficient at generating heat than the AuNS under 800 nm irradiation. It has been well-established that AuNS and AuNR generate heat by a plasmonic modality. Although HDAPPs generate heat by a different mechanism (recombination of bipolarons), the fact that they have similar PCEs to gold nanoparticles classifies them as good photothermal agents 10.

### Cytotoxicity and binding of HDAPPs *in vitro* and *ex vivo*

**Figure 2:**
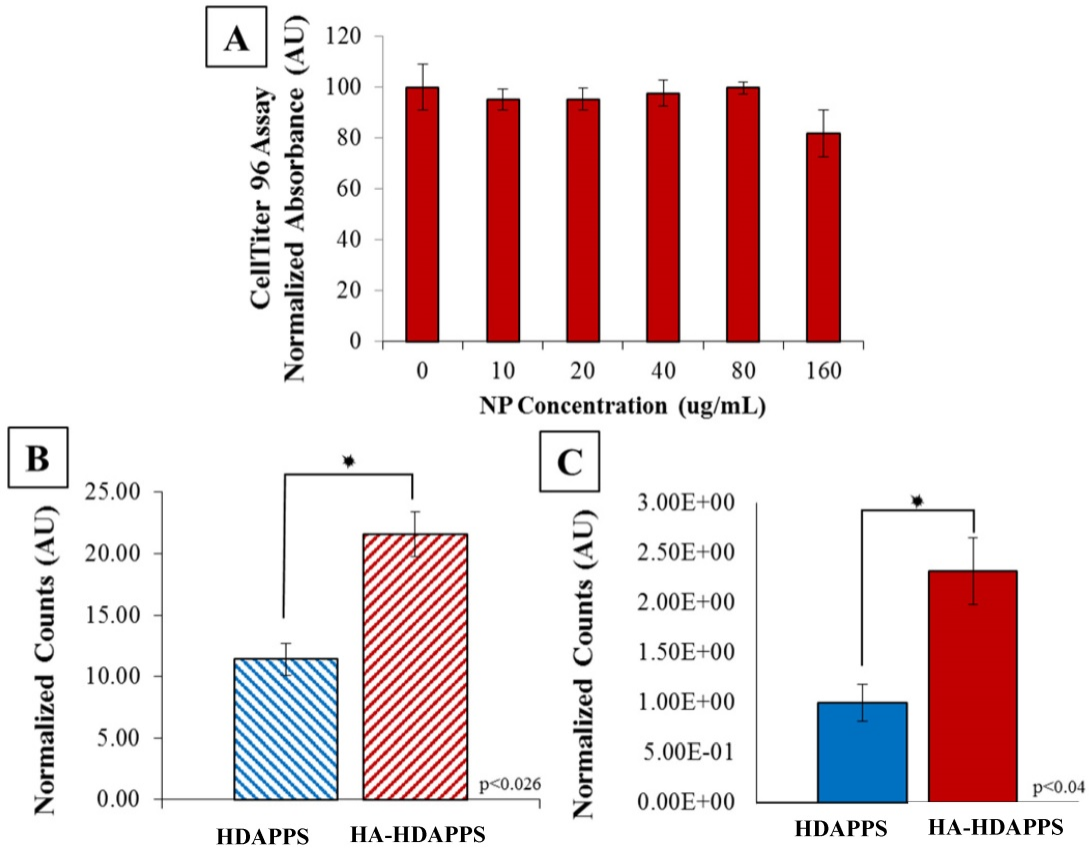
*In Vitro* Binding (**A**) CellTiter 96 assay of CT26 cells incubated with HA-HDAPPs for 24 hours at increasing concentrations. Cell viability was not significantly impacted at any concentration. (**B**) Graphical representation of percent bound material to CT26 cells *in vitro*, HA-HDAPPs bound 2-fold higher than HDAPPs. Error bars represent standard error of the mean. (**C**) Graphical representation of *ex vivo* binding of NPs to excised CT26 tumors. The fluorescence signal of tumors incubated with nanoparticles was blanked by subtracting tumor autofluorescence from saline incubated tumor controls. Error bars represent standard error of the mean.

To determine whether viability of the CT26 cells was affected by HA-HDAPPs, CT26 cells were incubated with various concentrations of HA-HDAPPs in culture media for 24 hours. The HA-HDAPPs showed no significant cytotoxicity to CT26 cells *in vitro* (Figure 2A), which corresponds to the findings with non-coated HDAPPs in previous works 10. As shown in Supplementary Figure 3A, there was no discernible difference in binding between the HDAPPs and HA-HDAPPs until the concentration surpassed 2 mg/ ml, which is a very high quantity of material. Furthermore, as shown in Supplementary Figure 3B, with laser stimulation, neither the HDAPPs, nor HA-HDAPPs that had bound to cells, with excess non-bound material washed away, had significant reduction in cell killing. However, when HA was functionalized to PCPDTBSe nanoparticles and exposed to near infrared stimulation, there was a significant reduction in cell viability, as seen in Supplementary Figure 3C. This effect was not observed with non-functionalized PCPDTBSe nanoparticles, which indicates that binding of the nanoparticles to the cells is critical for photothermal ablation. Although two dimensional binding indicates that a high concentration of HDAPPPs may be needed, the use of a 2D model may be misleading compared to three-dimensional or *in vivo* binding and we have seen excellent success with low concentrations of HDAPPs in a breast tumor model, hence an *in vivo* pilot trial was commenced.

In order to minimize the duration of mouse surgeries, the minimal amount of time needed for optimal nanoparticle binding was determined *in vitro* and was found to be 30 minutes, which also correlates with human HIPEC perfusions that take anywhere from 30- to 120-minutes 30,38. Binding of the HA-HDAPPs was assessed by a 30-minute incubation of the nanomaterials (non-coated HDAPPs and HA-HDAPPs) with CT26 cells. The fluorescence signal of the non-coated HDAPPs had the fluorescent intensity equivalent to ~11% bound and the HA-HDAPPs had fluorescent intensity signal corresponding to ~22% bound. Thus, the HA-HDAPPs had a two-fold higher binding than non-coated HDAPPs *in vitro* (p<0.026) (Figure 2B).

Tumors removed during surgery of the saline control mice were used for *ex vivo* binding experimentation to compare the binding efficiency of HA-HDAPPs to the non- functionalized HDAPPs. Bioluminescence was used to confirm that the excised tumors (~19 mg each) were composed of CT26 cancer cells and the fluorescence signal of the nanoparticles bound to the respective tumors was determined using IVIS. The fluorescence signal of the HDAPPs and HA-HDAPPs was compared using a Students t-Test. The total fluorescence signal difference between the tumors incubated with nanoparticles was determined by subtracting the tumor autofluorescence of the saline tumor controls and was 2.3 fold higher (p<0.04) in the HA-HDAPPs than the HDAPPs (Figure 2C).

### Excised Murine Disseminated Tumors

**Figure 3:**
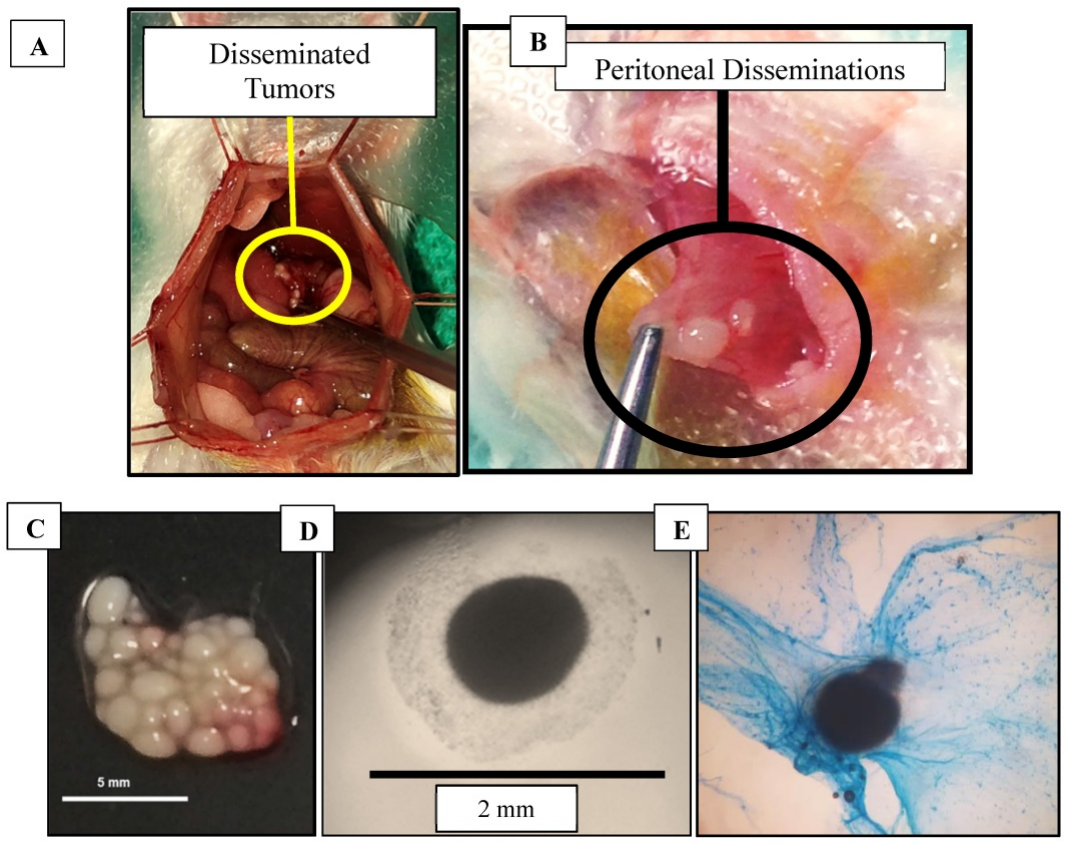
Tumors (**A**) Colorectal cancer disseminated tumors in mouse model during surgery, (**B**) Peritoneal-seeded tumors, (**C**) Excised tumors in a 100 mm dish on a black background, (**D**) Excised tumor secreting mucus imaged using a bright field microscope, (**E**) Tumor stained with Alcian blue to detect mucopolysaccharides.

Mice that were injected with CT26 cells developed both non-adherent and adherent disseminated tumors within the peritoneum (Figure 3A,B). The disseminated tumors were easily removed by saline flushes or by physical manipulation with cotton swabs. Larger tumors (>2 mm) that were adhered to the peritoneal cavity had to be removed with bipolar cauterizers to minimize bleeding. Some of the collected tumors were found to secrete mucus, which was confirmed by Alcian blue staining (used to identify mucopolysaccharides) (Figure 3C-E).

### Comparing HA-HDAPPs Binding *in vivo* using Perfusion versus Intraperitoneal Injection

After mice recovered from surgery, they were returned to their respective cages overnight, and then imaged again to determine nanoparticle co-localization to disease (Supplementary Figure 4). The mice were survived out to a humane endpoint after surgery in order to (1) verify that the mice were not succumbing to complications from the surgery and (2) demonstrate that the nanoparticles remained bound to the tumors. No fluorescence was observed in mice treated with saline, with and without lasert stimulation, as shown in Supplementary Figure 5. Laser alone, with the saline, did not reduce the tumor burden. The co-localization of nanoparticles to the remnant tumors was difficult to confirm with certainty in live mice using IVIS. Since the disseminated tumors are scattered throughout the abdominal cavity, whole body bioluminescence images from IVIS gives limited insight to the exact location of tumors with respect to the nanoparticles. It was observed that in the mice that received the HA-HDAPPs perfusion and the perfusate collected, that the perfusate was entrapping free-floating cells and tumors (Supplementary Figure 6). It was hypothesized that, by injecting the HA-HDAPPs 24 hours prior to surgery on the mice, the HA-HDAPPs would have more time to bind to tumors or possibly become entrapped in the secreted mucus. Indeed, tumors that were removed from these mice during surgery were found to have a bronze film, where IVIS imaging confirmed that these tumors had nanoparticles bound. This result may also be due to the possibility that these nanoparticles are becoming entrapped in the mucus.

### *In vivo* Photothermal Ablation

There was wide variability of the tumor burden following either nanoparticles delivered via perfusion, or IP injection with and without laser. As demonstrated by Supplementary Figures 7-10, only mice that received IP injection plus laser (Supplementary Figure 10) had a reduction in the tumor burden. However, this group also had an animal with an increased tumor burden over time. The application of HA-HDAPPs delivered via perfusion and laser was not successful in reducing the tumor burden (Supplementary Figure 8). However, this failure may be partially due to the rapid spread of the micro-tumors in this animal model. One of the major challenges with photothermal ablation in the mouse model of PC of CRC is that the blood and feces both absorb sufficient 800nm light to cause non-specific heating and coagulative necrosis of the small blood vessels, duodenum and intestines. Insufficient binding of the perfusion delivered HA-HDAPPs, non-specific heating, and the inability to evenly distribute the laser light in the murine model limits further evaluation of the photothermal ablation for PC of CRC in a murine model. Such limitations might not be as challenging in a larger animal model, especially if fasting can be utilized to eliminate the fecal material in the intestines that also absorbs NIR. The perfusion technique (Supplementary Figures 7 and 8) indicates some dispersion of the HA-HDAPPs and association with the CT26 tumors. And, there is a reduction in the HDAPPs signal following laser stimulation, which indicates nanoparticle clearance. The IP injection delivery shows that there is minimal reduction, and possibly a slight increase, in the HDAPPs fluorescence intensity (mouse 2 in Supplementary Figure 9 and all three mice in Supplementary Figure 10). Even more interesting is that the HA-HDAPPs delivere3d by IP injection, and then stimulated with NIR, remain, even with the resolution of the tumor. It is possible that the HA-HDAPPs are remaining bound to the peritoneum following photothermal therapy and clearance or the tumor cells, and was an unexpected result of the pilot study.

**Figure 4:**
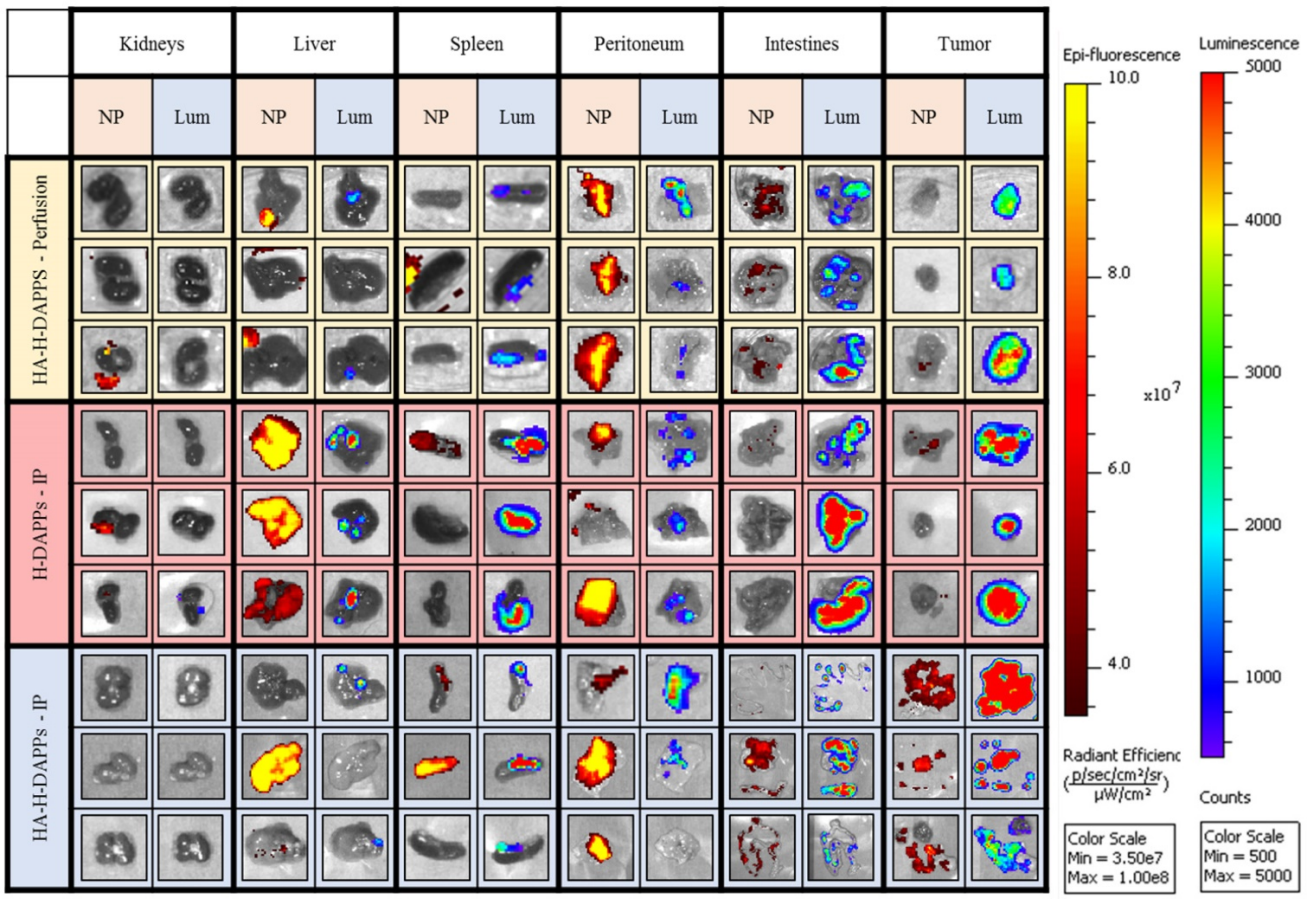
Comparing Nanoparticle Co-localization to Tumor Areas in the Excised Organs Using Various Conditions/Nanoparticles - Three mice per group (HA-HDAPPs delivered via perfusion, HDAPPs delivered via IP injection, HA-HDAPPs delivered via IP injection), with six tissues per mouse (TOP - kidneys, liver, spleen, peritoneum, intestines, and excess tumor). The nanoparticle signal is indicated by the red-yellow colors and the cancer bioluminescence signal denoted by Blue, Green, Red.

This murine colorectal cancer model produced a high number of small tumors disseminated throughout the abdominal cavity with no evidence of metastasis outside of the peritoneal cavity. Once the animals were euthanized and the organs were excised, co-localization of disease and nanoparticles was easier to distinguish with IVIS (Figure 4). The individual organs were imaged for nanoparticle fluorescence with IVIS, and then luciferin was added to the organs to induce the bioluminescence signal of the CT26 cells to distinguish tumors from non-cancerous tissue. The bioluminescence from the intestines indicates seeding of CT26 cells to these tissues, which was often observed during surgical debulking. One interesting observation was that mice treated with IP injection of the nanoparticles had an increase in fluorescence in the tumor over time, indicating that perhaps a longer time-point than 24 hours is needed to maximize binding to micro-tumors. Some tumor was observed in the liver, intestines, spleen and peritoneum. There was a significant correlation with the HDAPPs fluorescent signature in the spleen and liver, but only for nanoparticles delivered by IP injection. HA-HDAPPs delivered via IP injection were best localized to tumor nodules. All HDAPPs types and delivery led to an association with the peritoneum, possibly due to the presence of micro-metastatic lesions.

Nanoparticle binding on the excised tumors and tissues was qualitatively evaluated for HA-HDAPPs perfusion delivery compared to delivery by IP injection of HDAPPs or HA-HDAPPs (Supplementary Figure 11). Nanoparticles were not observed in the brain or heart in any of the nanoparticle mice; minimal nanoparticle binding was observed in the lungs of an H-DAPPs IP injection treated mouse and in the kidneys of one HA-HDAPPs perfusion mouse. High nanoparticle signal was observed in the livers and spleens of several mice that received nanoparticles via the IP injection; however, this was less evident in the HA-HDAPPs IP mice. Highly dense tumor regions on the peritoneum and intestines in the HA-HDAPPs groups showed moderate to high nanoparticle signal. These results are very encouraging for supporting the binding affinity of the HA-HDAPPs to the disseminated cancer. However, not all of the disseminated tumors removed from the mice had bound nanoparticles, which is most likely due to post-operative development of new tumors that were not exposed to the nanomaterials during the initial delivery. Quantification of the amount of nanoparticles in each respective tissue type was attempted by ICP-MS; however, these results were not significant due to the high amount of selenium in native tissue (Supplementary Figure 12).

## Conclusions

Disseminated CRC remains one of the top contributors to cancer related deaths worldwide and the development of better disease models and therapeutics to treat this disease are necessary. The versatility of the established immunocompetent BALB/c mouse model with its syngeneic colorectal cancer line CT26 was used to develop a disseminated colorectal cancer model to mimic human disease. The murine dissemination model stressed the difficulty in detecting micro-tumors and focused on tumor number rather than tumor size. Surgeries for CRC are focused on removing as many lesions as possible while simultaneously trying to spare as much healthy tissue as possible. However, this is exceedingly difficult because the disseminated disease makes it difficult to distinguish tumor-contaminated tissue from non-tumor-infiltrated tissue. In this work, we demonstrated that hyaluronic acid can be attached to fluorescent nanoparticles to aid in their targeting to disseminated micro-tumors of CRC. The HA-HDAPPs used here have a fluorescent signature which demonstrates their ability to highlight the tumors for subsequent photothermal treatment. HA-HDAPPs delivered via peritoneal perfusion or IP injection both had localization to the tumor, but only mice treated with IP injection and laser had any regression of the tumor burden.

Advances in the field of nanomedicine are bridging the gap between clinical challenges and improved clinical diagnostics and outcomes 7,40-42. HDAPPs are classified as theranostics, materials that perform both as a therapeutic and an imaging agent for tumor visualization. Here, the adaptability of HDAPPs was exemplified by the addition of a targeting component, hyaluronic acid (HA). HA-HDAPPs were successfully prepared using a facile coating method and were shown to have similar properties to the core material. The addition of HA increased the binding of the HDAPPs *in vitro, ex vivo* and *in vivo* to CT26 murine colorectal cancer cells that expressed CD44. HA-HDAPPs were delivered via an open abdominal perfusion or an intraperitoneal injection, where both methods show co-localization of nanoparticles to tumor regions based on the NIR fluorescence signature of the nanoparticles. One of the key advantages of the HDAPPs formulation is the fluorescent signature from the PFBTDBT10; this allows for visualization of the nanoparticles co-localized with the CRC micro-tumors. Although fluorescence is advantageous, the 20: 1 ratio of PFBTDBT10 to PCPDTBSe in the HA-HDAPPs generate good bulk heating but do not cause significant cell death in two dimensions, even with good binding. This is due to the low amount of the heat generating PCPDTBSe polymer in the HDAPPs formulation. Although it seems straightforward to simply add more of the heat generating polymer, such a modification limits the fluorescence because the PCPDTBSe can absorb the emitted photons, leading to quenching. The current limitation may be overcome in future iterations of the HDAPPs through precision structuring of the polymer chains within the nanoparticle to minimize this phenomenon. Alternatively, delivery of a solution composed of HA-PCPDTBSe and HA-HDAPPs could aid in enhanced *in vivo* photothermal destruction. These results presented here highlight the potential for using HDAPPs functionalized with hyaluronic acid to target and photothermally treat disseminated CRC.

## Supplementary Material

Supplementary figures and information.

## Figures and Tables

**Table 1:** Hydrodynamic diameter and zeta potential of HDAPPs with chitosan then hyaluronic acid coatings

Nanoparticle	Diameter (nm)	PDI	Zeta Potential (mV)
HDAPPs	82.8 - 102	0.118 - 0.179	- (13.2 - 26.8)
Chitosan Coated	94.1 - 152	0.180 - 0.213	1.3 - 24.5
Hyaluronic Acid Coated	172 - 242	0.203 - 0.350	-_(19.7 - 52.3)
