# Supplementary material for: Binding of Targeted Semiconducting Photothermal Polymer Nanoparticles for Intraperitoneal Detection and Treatment of Colorectal Cancer
Source: Nanotheranostics. 2020 Mar 26;4(3):107–18. doi: 10.7150/ntno.29522 (PMC7171385; doi:10.7150/ntno.29522)
Supplement: Supplementary file 1 — Supplementary figures and information. [file ntnov04p0107s1.pdf]

Supplementary Information

Photothermal Conversion Efficiency

Photothermal conversion efficiencies (PCE) of HA-HDAPPs, HDAPPs and BSe NPs were determined by irradiating 3.9 mL of 50 µg/mL solutions of respective nanoparticles in a 10 mm path length quartz cuvette (QS High Precision Cell, 100-10-40). The PCEs of these nanoparticles were compared to gold nanoshells (AuNS) (1.8 µg/mL, NanoComposix, JMW1612) and gold nanorods (0.05 OD_{800nm}, NanoComposix, BAM0182). Nanoparticle solutions under continuous stirring were exposed to 800 nm laser (1 cm diameter, 3W) for 60 minutes while temperature was monitored at a 1 second sampling rate with a Neoptix NOMAD-*Touch* Optical Thermometer. Following irradiation, the solution temperatures were monitored until the initial, ambient temperature (T_{amb}) prior to irradiation was achieved. To determine the characteristic time constant for cooling, the data was truncated to avoid noise within the thermometer for when the temperature had cooled to between 6-11% of the maximum temperature difference. Photothermal conversion efficiency was calculated according to a previously established protocol.[31,32] First, the characteristic time constant for cooling (τ_s) was determined by plotting the non-dimensionalized temperature driving force (Eq. 1) vs. time and taking the negative reciprocal of the slope of a linear fit of the data.

$$\theta = \frac{T_{amb} - T}{T_{amb} - T_{max}} \qquad \text{[Eq.1]}$$

To determine the heat loss due to external heat flux, the linearized form of Newton's Law of Cooling is employed as in equation 2.

$$Q_{ext} = hA(T - T_{amb}) \qquad \text{[Eq. 2]}$$

To solve for the heat-transfer coefficient across the entire area of flux (hA), equation 3 is utilized.

$$\tau_s = \frac{\sum_i m_i C_{p,i}}{hA} \qquad \text{[Eq. 3]}$$

Under the assumption that the nanoparticles are a negligible component of mass ($m_{np} < 0.005$ % total mass), the system properties are defined for water with a heat capacity (C_{p,H_2O}) of 4.18 kJ/kg*K and a

total mass of 3.9 g. The hA term is then utilized to solve the photothermal conversion efficiency at steady state laser irradiation, where the heat dissipation due to water absorption (Q_O) was determined to be 0.0692 W and subtracted from the overall heat generation according to equation 4. Q_I is the energy influx due to nanoparticle absorption and is described by equation 5, where I is the laser irradiation power, A_λ is the absorption of nanoparticles within the cuvette system at 800 nm, and η_T is the photothermal conversion efficiency.

$$Q_I = hA(T - T_{amb}) - Q_O \quad \text{[Eq. 4]}$$

$$Q_I = I(1 - 10^{-A_\lambda})\eta_T \quad \text{[Eq. 5]}$$

Immunofluorescence

CD44 expression was determined by immunofluorescence microscopy. CT26 cells were grown on poly-L-lysine coated glass slides and fixed with 4% paraformaldehyde for 10 minutes, then rinsed three times with 1 x PBS. Cells were permeabilized with 0.1% TritonX-100 for 10 minutes then rinsed three times with 1 x PBS. Cells were blocked with 1% BSA (bovine serum albumin) for 30 minutes, rinsed with 1 x PBS, then incubated with anti-CD44 (Abcam - ab157107, rabbit polyclonal) at 1:1000 in 1% BSA at 4°C overnight. The next day the primary antibody was removed and the cells were rinsed three times with 1 x PBS. The secondary antibody, goat-anti-rabbit Alexa 488 (Abcam ab150077) was incubated 1:1000 in 1% BSA for one hour at ambient temperature for 1 hour in the dark, then the cells were rinsed three times with 1 x PBS. A 1:1000 DAPI stain was incubated on the cells for 60 seconds then rinsed three times with 1 x PBS. Cells were imaged on an Olympus IX70 epifluorescence microscope.

In Vivo Bioluminescence

D-Luciferin (200 µL of 15 mg/mL stock; PerkinElmer) was injected IP into the mice 24 hours after implantation, 12 hours post surgery, and every 48-72 hours. The bioluminescence signal was

detected using an In Vivo Imaging System (IVIS: Caliper-PerkinElmer) with 1 and 10 second exposures on the front and back sides of the animals, using subject heights of 1.5 cm, and medium binning. The Living Image Software (PerkinElmer) was used to quantify the total bioluminescence signal (photons/second) of the disease in the animals. Animals were considered at endpoint when, using IVIS, the bioluminescence signal saturated at 1-second exposure.

Nanoparticle Perfusate Analysis

HA-H-DAPPs solutions that were perfused in mice were collected after every surgery. The nanoparticle perfusates were transferred to a 35 mm dish and observed using a light microscope with a 10x objective. The same perfusates were imaged using IVIS and excited at 465 nm to image nanoparticle fluorescence. Finally, 150 µg/mL D-luciferin was added to the perfusate to image for CT26 cells' bioluminescence.

CD44 Detection in CT26 Murine Cells

CD44 expression in CT26 cells was confirmed by Western blotting and immunofluorescence. Western blot showed low staining at the predicted MW of 81 kDa for CD44 in CT26 cells, but had strong bands at lower molecular weights. This could be due to aberrant glycosylation of these cells. The same antibody was used for immunofluorescence staining and verified the presence of CD44 in CT26 cells. (Supplementary Figure 1)

Perfusion Solutions

HA-H-DAPPs stocks were stable in saline for a few days and did not precipitate or aggregate during the 30 minute perfusion in the mouse abdomens. However, shortly after the HA-H-DAPPs perfusate was collected following the abdominal perfusions, the nanoparticles always precipitated out of solution (Supplementary Figure 5A). Interestingly, non-functionalized H-DAPPs in saline that were

perfused in a mouse for 30 minutes never aggregated or precipitated after being collected (Supplementary Figure 5B). The HA-H-DAPPs perfusates were observed using a light microscope with 100x magnification. It was perceived that the brown precipitated aggregates (which were assumed to be aggregated HA-H-DAPPs) had entrapped cells and possibly small disseminated tumors (Supplementary Figure 5C&D). In order to verify the identity of the aggregations, cells, and tumors in the perfusates, the HA-H-DAPPs perfusate was imaged for nanoparticle fluorescence and CT26 bioluminescence using IVIS to confirm that the brown aggregates in the HA-H-DAPPs perfusate were comprised of the nanoparticles (Supplementary Figure 5E). A few drops of luciferin were added to the aggregates in the perfusate and bioluminescence signals were observed, indicating that CT26 cells and tumors were entrapped in the aggregates (Supplementary Figure 5F). (Note that the aggregates in the perfusate slightly shifted their location in the image upon addition of the luciferin.)

Ex Vivo Staining

Disseminated tumors removed from saline control mice during surgery (<2 mm) were rinsed with saline and fixed with ethanol on microscope slides. The tumors were briefly stained (10 minutes) with 0.05% Alcian blue stain to stain negatively charged mucins. The tumors were rinsed with saline and then covered with a coverslip and imaged with a 10x objective using a light microscope.

Inductively Coupled Plasma Mass Spectrometry (ICP-MS)

In order to quantify the total amount of nanoparticles bound to, or cleared to, specific tissues and tumors, Inductively Coupled Plasma Mass Spectrometry (ICP-MS) was used to detect the selenium in the PCPDTBSe polymer component of the H-DAPPs. After imaging, all tissues were rapidly frozen at -80°C and stored until further use. Next, the tissues were weighed, lyophilized, re-weighed, and stored in a desiccator to prevent moisture contamination prior to preparation for ICP-MS analysis. The dried tissues were then microwave digested using 2 mL of nitric acid (TraceMetal Grade, Fisher Scientific), 3

mL of 30% hydrogen peroxide (Low Trace Metals, GFS Chemicals) and 5 mL of Milli-Q water using an Ethos Up Microwave-Assisted Digestion system (Milestone). Samples were diluted to 50 mL using Milli-Q water to yield 4% nitric acid solutions and stored in 50 mL conical tubes at room temperature. A calibration curve with selenium standards was made using 0, 0.1, 0.2, 0.5, 1.0, 2.0, 5.0, 10.0, and 20.0 ppb selenium in a 4% nitric solution. Samples were run on an Agilent 8800 ICP-MS/MS to detect the selenium concentration in each sample. Selenium concentration in each respective tissue was calculated as Se in µg/g of tissue. (Supplementary Figure 12). H-DAPPs have a selenium component that accounts for approximately 0.675% of the total weight of the polymer blend (5% PCPDTBSe to 95% PFBTDBT10). This was determined by estimating that selenium makes up ~13.5% of the PCPDTBSe complex by mass, but since PCPDTBSe makes up only 5% of the H-DAPPs complex (13.5% * 5%), the selenium component is less than 1% of the H-DAPPs. Therefore, the amount of bound nanoparticles in each respective tissue from mice in the different treatment groups (saline perfusion, nanoparticle perfusion, IP nanoparticle delivery) could be quantified. Note that tumors and intestines were grouped as one tissue because tumors were mainly integrated on the intestines and were isolated as one "tissue". The amount of selenium in each tissue type was calculated as micrograms of selenium per gram of tissue. A one-way ANOVA was used between the different surgery types (saline perfusion (n=3), nanoparticle perfusion (n=4), and IP nanoparticle delivery (n=4)) for each specific tissue type.

The data collected here did not show statistically significant amounts of selenium between mice in the saline perfusion or the nanoparticle treatments in any tissue (Supplementary Figure 12). The trend in the data shows that there is more overall selenium in the nanoparticle surgeries, as expected, and the most selenium is in the IP nanoparticle surgeries. It is important to note that the nanoparticle concentrations in the respective tissues among the different treatment groups were not found to be significant based on the ICP-MS quantification data. The ICP-MS data did illustrate trends that more selenium was in tissues that had higher fluorescence signals from IVIS. Although this was a pilot study and the treatment groups were small, it is possible that there was not adequate power for the statistical

analysis.

Supplementary Figures

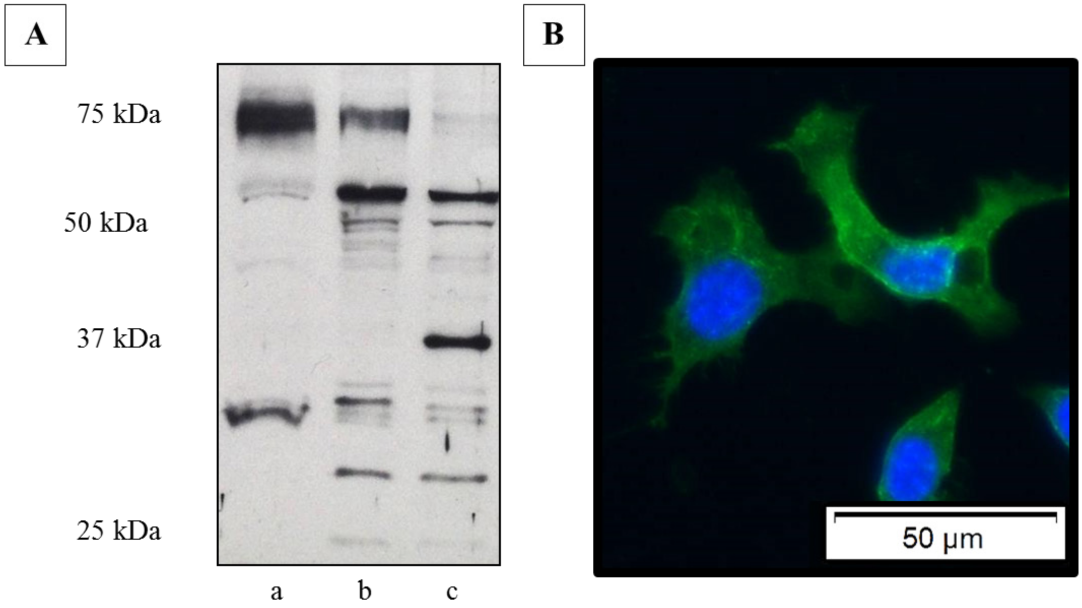

Supplementary Figure 1. (A) Western blot of CD44 staining in (a) NIH-3T3, (b) CMT-93, and (c) CT26 cells. (**B**) Immunofluorescence staining of CD44 in CT26 cells using anti-CD44 antibody (1:1000) (green) and a DAPI (blue) nuclear stain.

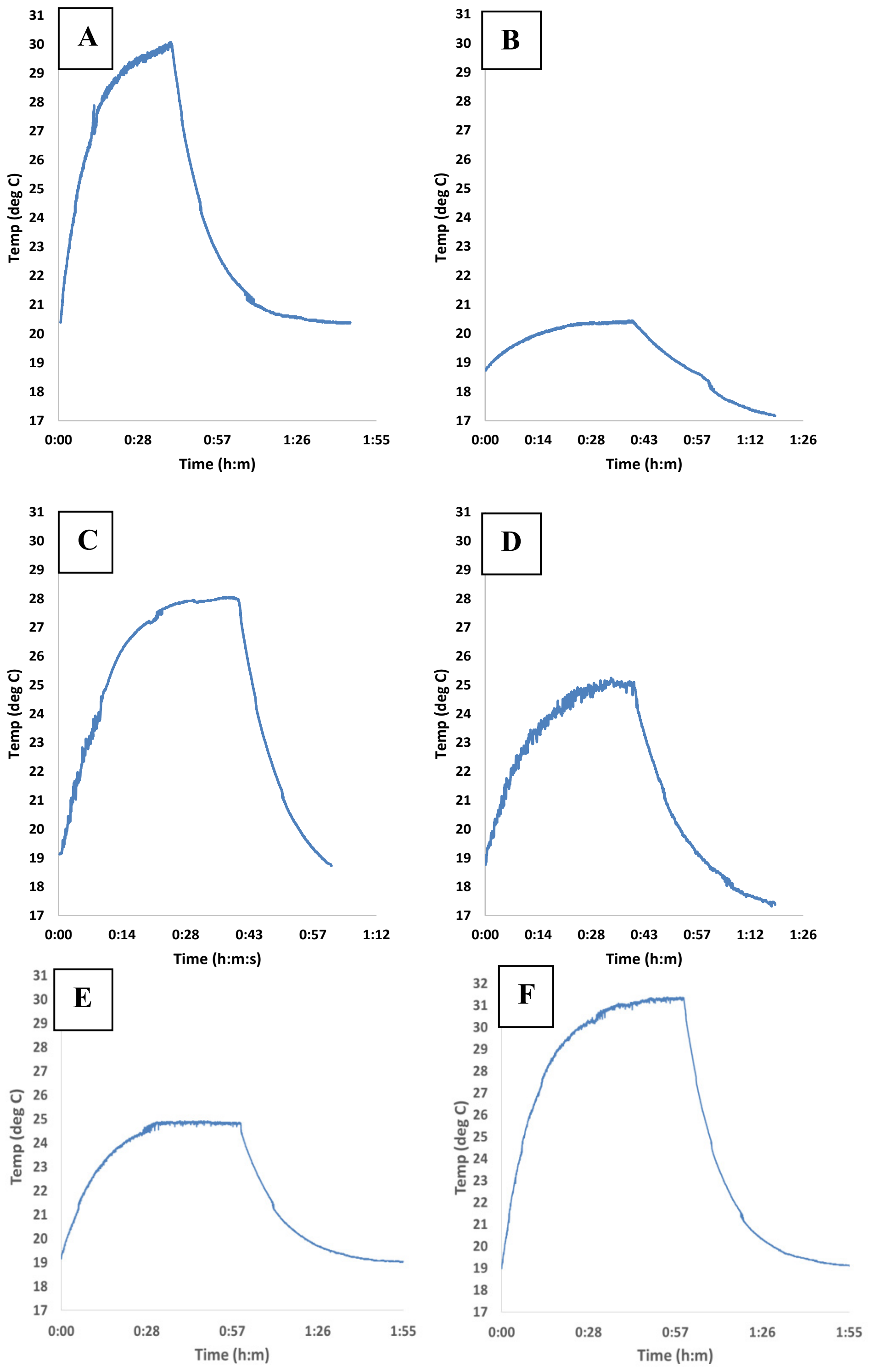

Supplementary Figure 2. Photothermal efficiency curves for **(A)** PCPDTBSe nanoparticles, **(B)** Water, **(C)** HDAPPs, **(D)** HA-HDAPPs, (**E**) gold nanoshells and (**F**) gold nanorods.

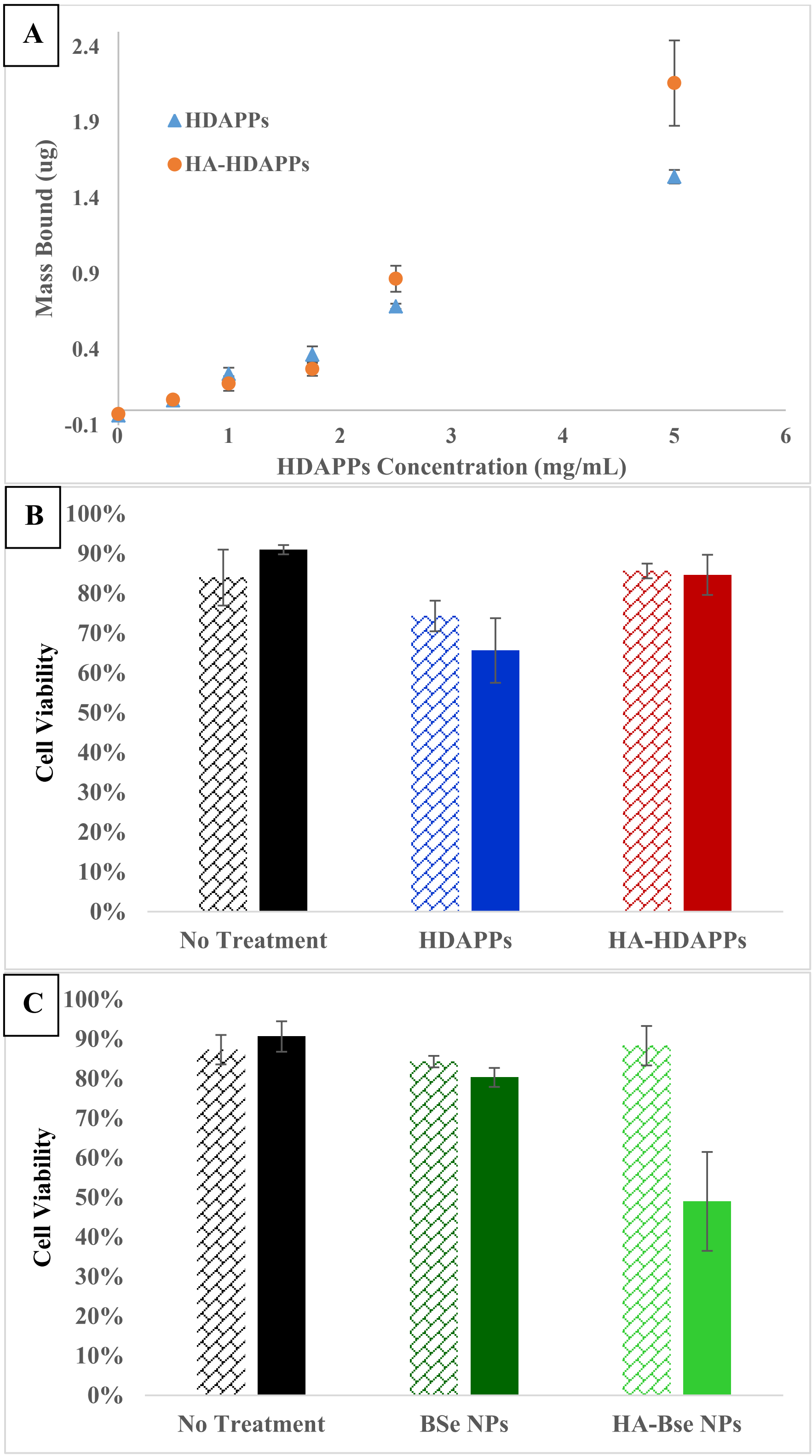

Supplementary Figure 3. (**A**) Binding of HDAPPs and HA-HDAPPs onto CT26 cells for 1hr at 4°C. Cell viability of CT26 cells treated with HDAPPs (**B**) or PCPDTBSe NPs (**C**). Solid bars indicate no laser stimulation and hashed bars indicate that 180 J/cm^2 was applied.

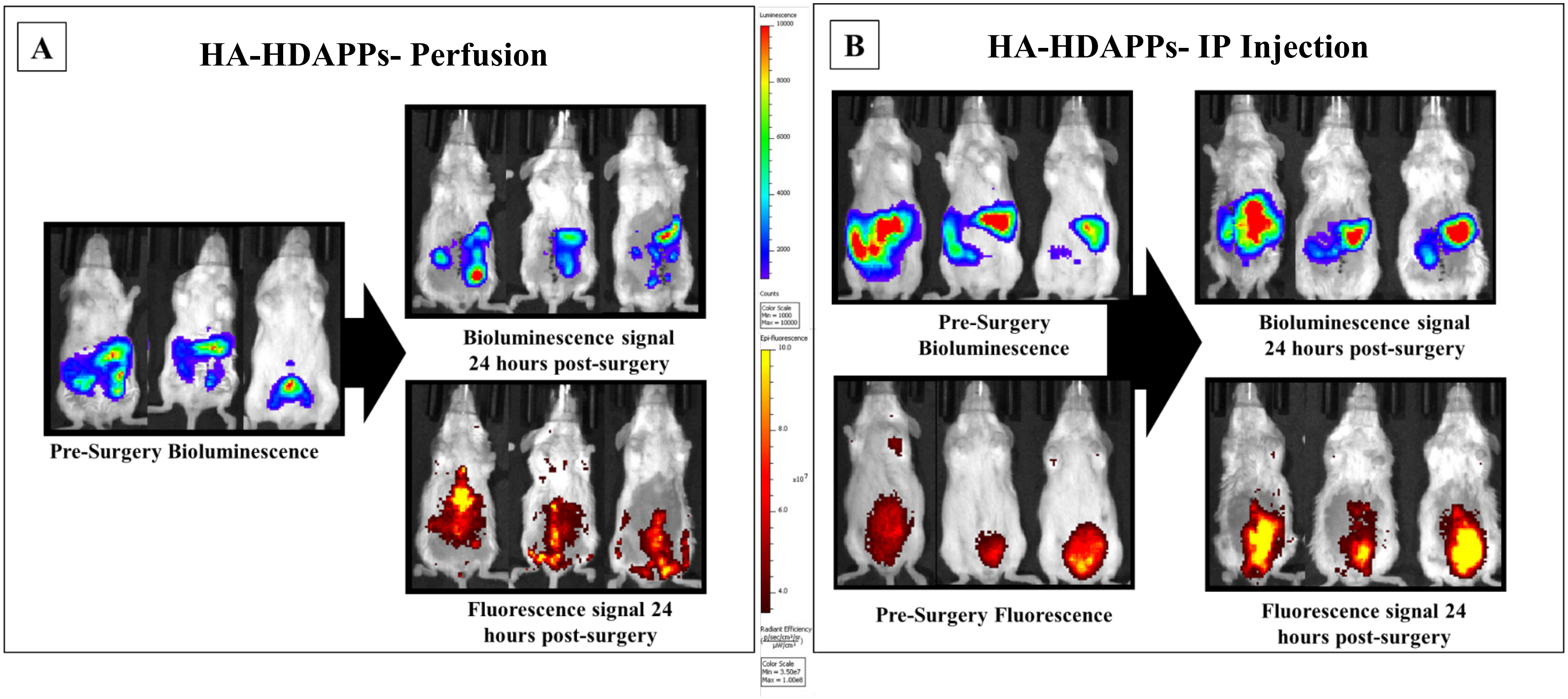

Supplementary Figure 4. HA-HDAPPs Co-Localization Post Perfusion and Via IP Injection. (**A**) Post-surgery fluorescence of HA-HDAPPs and cancer cell bioluminescence in mice 24 hours following perfusion/debulking surgery. (**B**) Post-surgery fluorescence of HA-HDAPPs and bioluminescence in mice 24 hours following IP injection, and 24 hours following surgery and saline rinse.

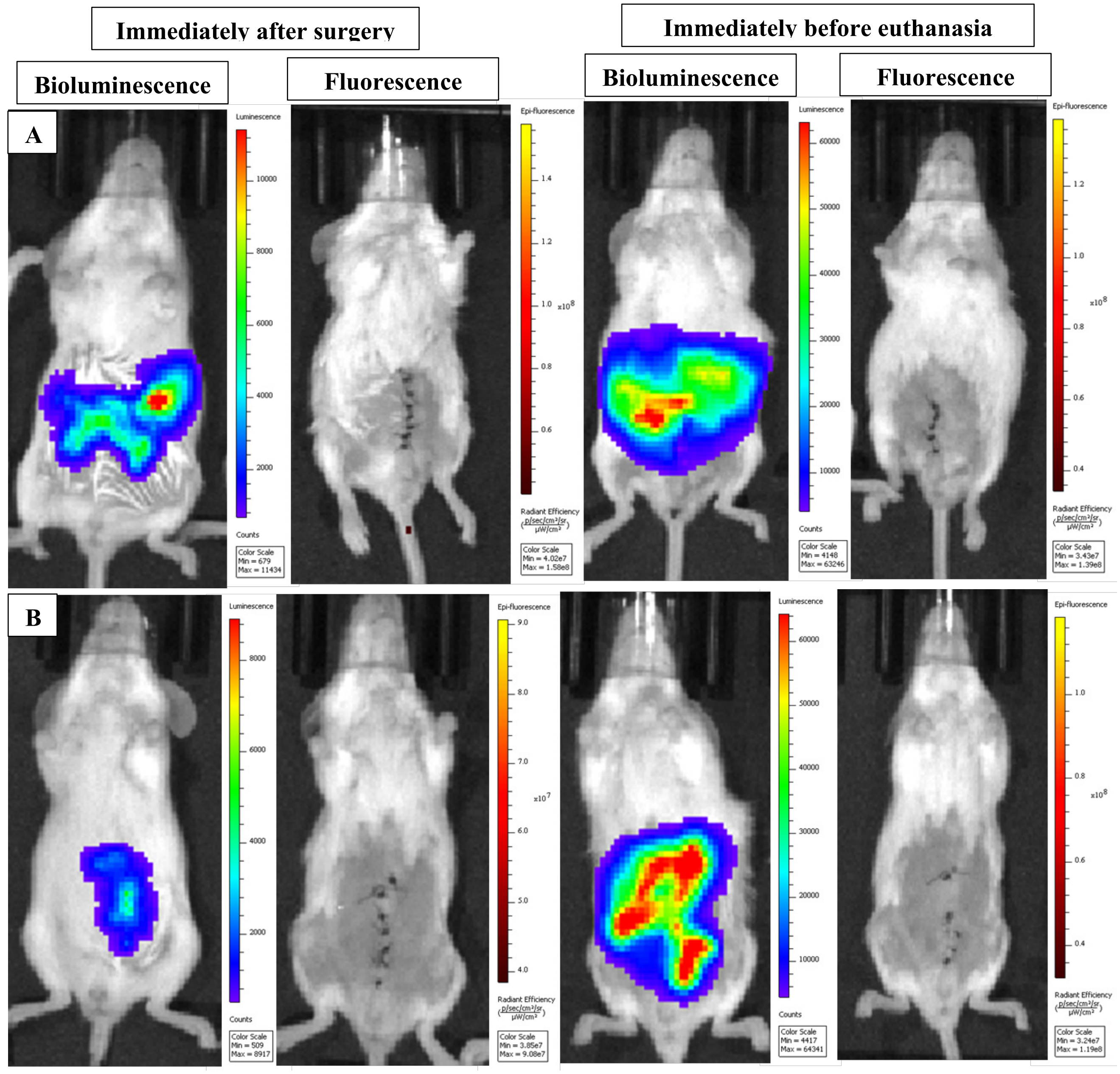

Supplementary Figure 5. One mouse in each representative group treated with macroscopic tumor debulking and saline perfusion with (**A**) and without (**B**) laser (180 J/cm^2) stimulation. The black dots along the midline are closure sutures. Tumor burden is indicated by the blue/ red images (bioluminescence). As expected, there is no red/ yellow fluorescence seen, as no HDAPPs were delivered.

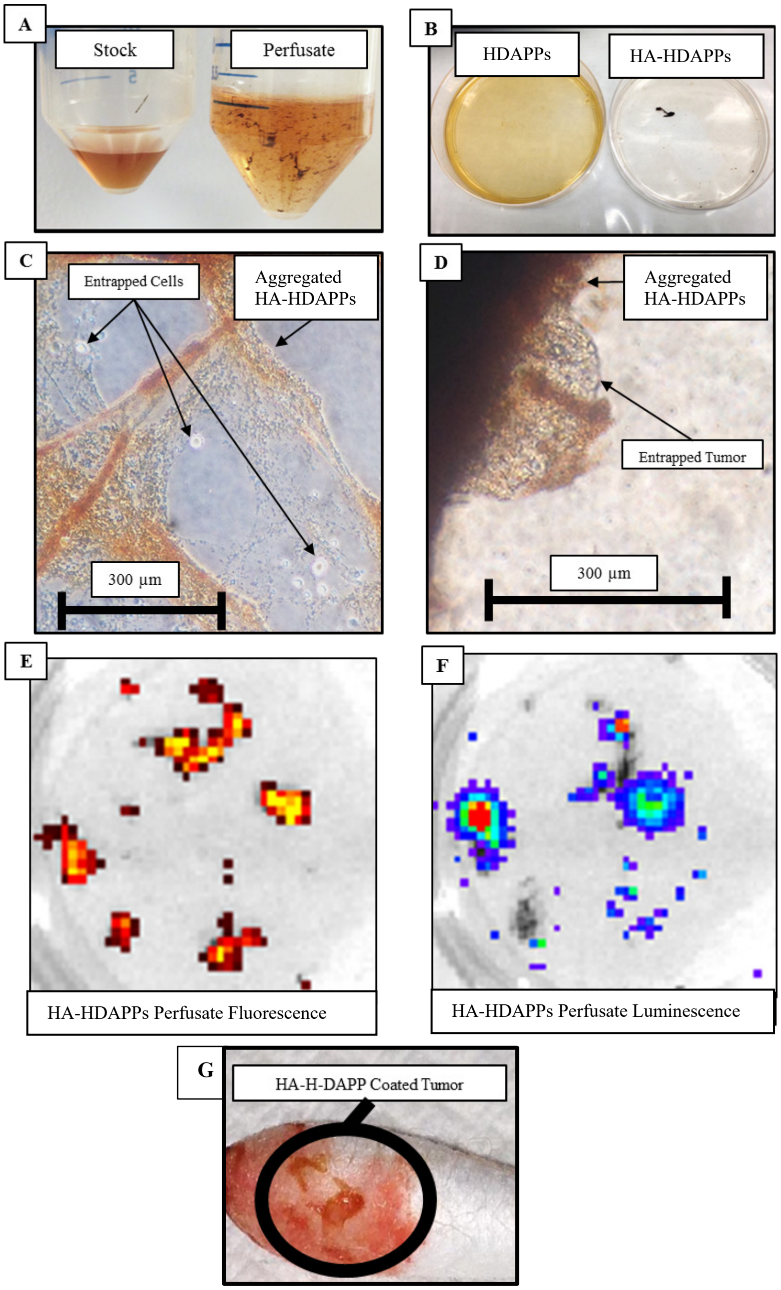

Supplementary Figure 6. (**A**) Stock HAH-DAPPs solution (**LEFT**) and HA-HDAPPs perfusate collected from a mouse perfused with the stock HA-HDAPPs for 30 minutes (**RIGHT**). The HA-HDAPPs perfusate began to aggregate and precipitate out of solution shortly after collecting it from the mouse abdomen. (**B**) Comparison of HDAPPs (**LEFT**) and HA-HDAPPs (**RIGHT**) perfusates collected from two separate perfused mice. The HDAPPs perfusate did not aggregate but the HA-HDAPPs perfusate quickly aggregated. (**C**) Zoomed in light microscopy image of HA-HDAPPs perfusate with entrapped cells. (**D**) Zoomed in image of the same HA-HDAPPs perfusate with an entrapped tumor. (**E**) The same HA-HDAPPs perfusate was imaged for fluorescence by IVIS (465 ex, ICG filter) to confirm that the aggregates were nanoparticles. (**F**) The same HA-HDAPPs perfusate was treated with luciferin to illustrate that CT26 cells and tumors were entangled in the aggregates within the perfusate. The aggregates in the perfusate shifted upon addition of the luciferin to the 35 mm plate. (**G**) A cotton swab used to remove tumors during surgery has a tumor with a brown film, which is indicative of HA-HDAPPs.

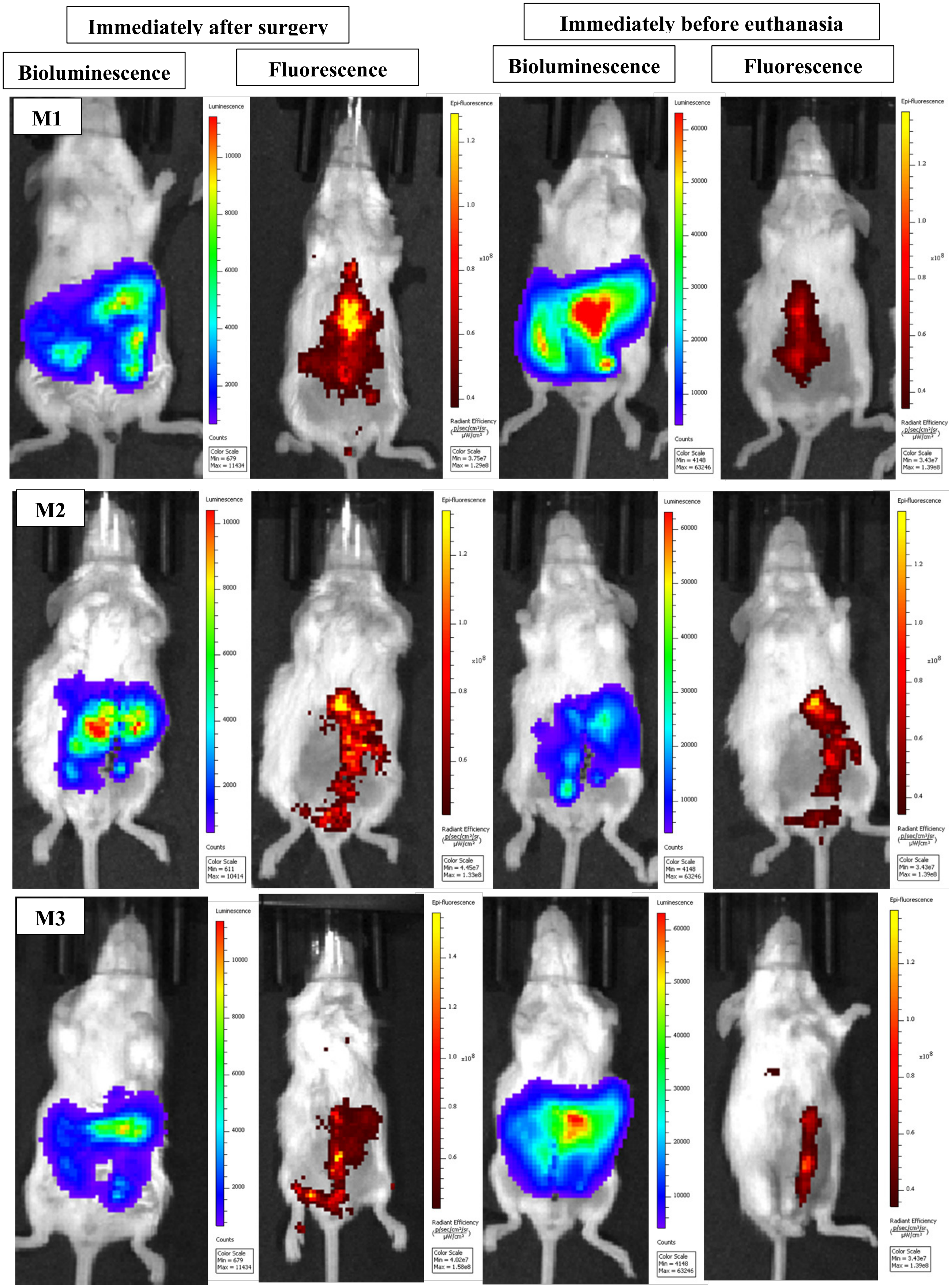

Supplementary Figure 7. Mice treated with HA-HDAPPS via perfusion and no laser stimulation.

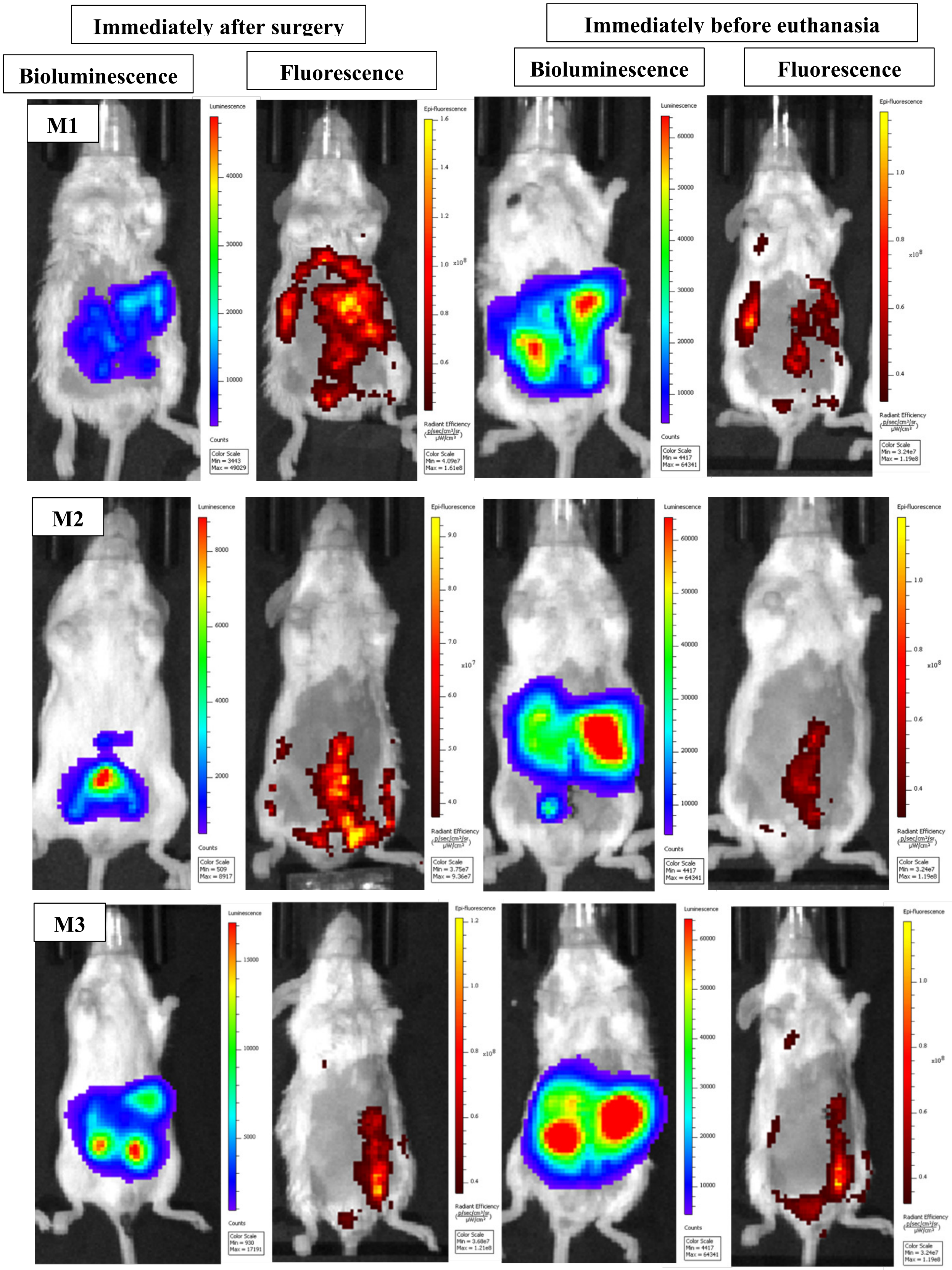

Supplementary Figure 8. Mice treated with HA-HDAPPS via perfusion and laser stimulation.

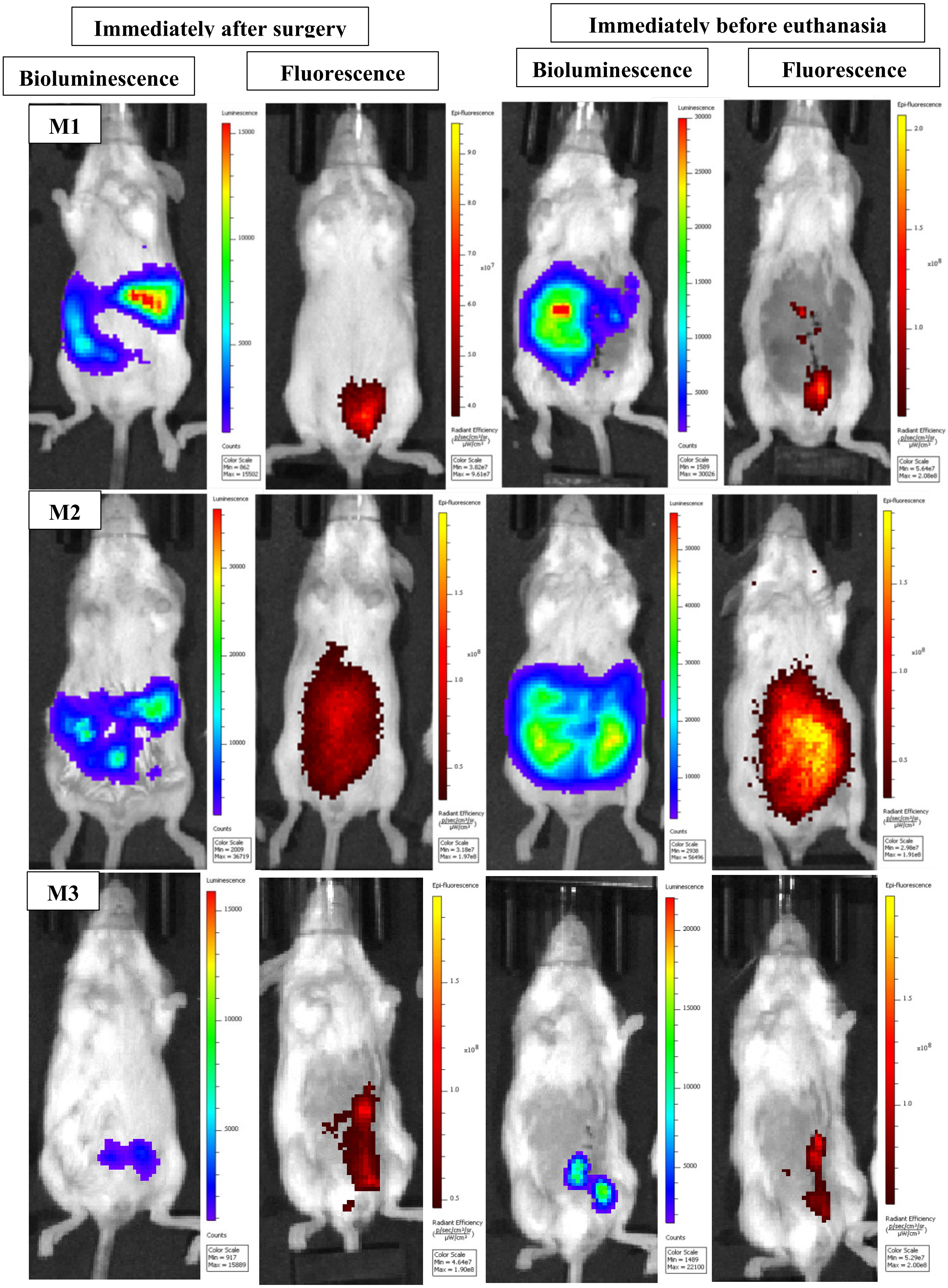

Supplementary Figure 9. Mice treated with HA-HDAPPS via IP injection and no laser stimulation.

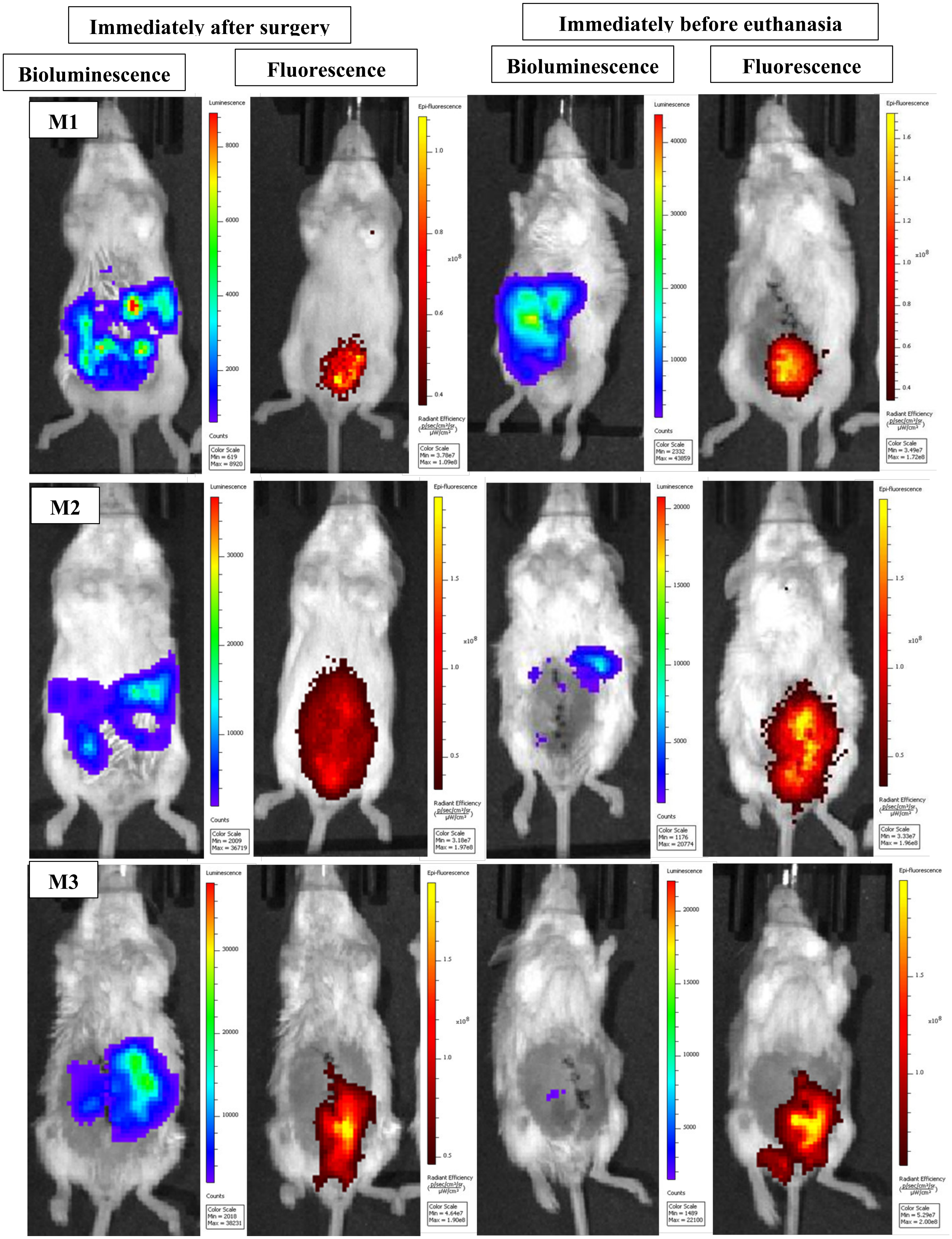

Supplementary Figure 10. Mice treated with HA-HDAPPS via IP injection and laser stimulation.

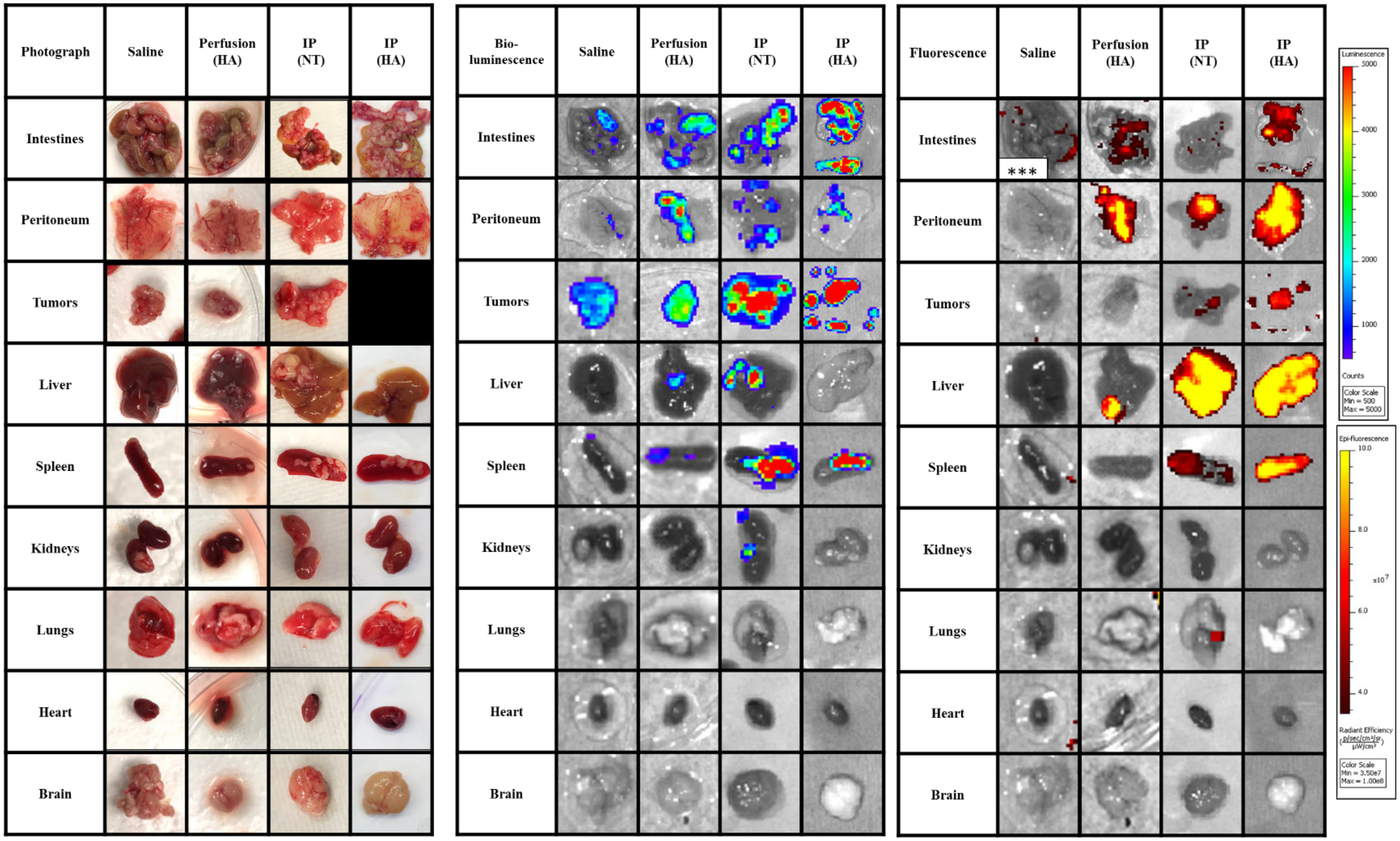

Supplementary Figure 11. Excised organs were evaluated to compare nanoparticle co-localization to regions of tumor. Photographs (LEFT) of the excised tissues were taken then they were imaged using IVIS to identify the luminescence of CT26 cells (CENTER) and nanoparticle fluorescence (RIGHT). Saline – Control Mouse, Perfusion (HA) – Mouse that received HA-HDAPPs via Perfusion, IP (NT) – Mouse that received non-targeted HDAPPs via IP injection, IP (HA) - Mouse that received HA-HDAPPs via IP injection. *** Note: In (RIGHT) Saline Intestines: The "nanoparticle" signal in the saline organs is from background signal from the tissue culture plastic.

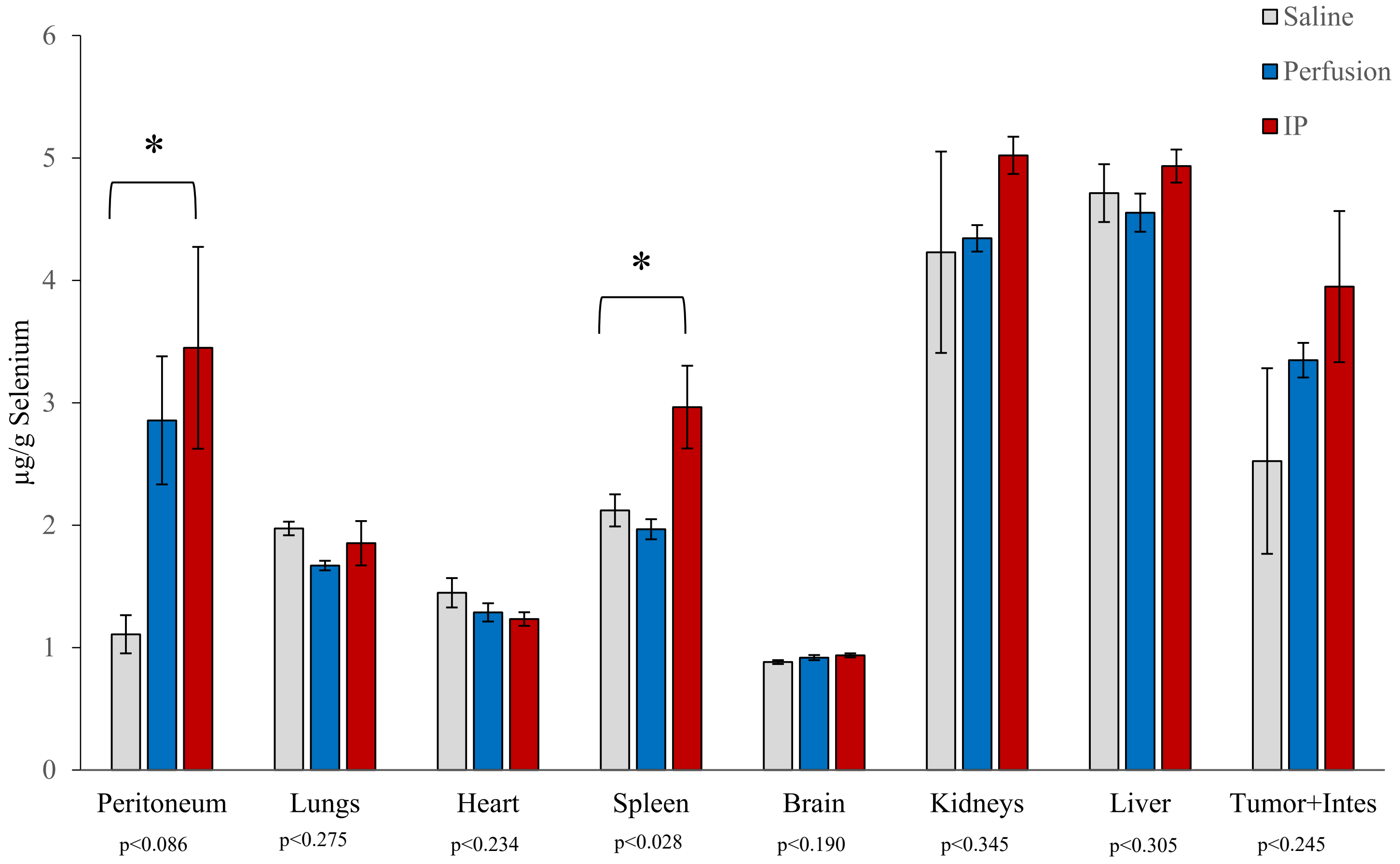

Supplementary Figure 12. ICP-MS quantification of selenium in tissues from HA-HDAPP (perfusion or IP injection methods of delivery) and saline treated mice. Selenium concentration was determined in each tissue in micrograms per gram of tissue. Selenium concentrations between the different groups in specific tissues were not significant (lung, heart, brain, kidneys, liver, tumor). There were statistically increased concentrations of selenium in the peritoneum of mice treated with HA-HDAPPs delivered by perfusion or IP injection. There was also a significant increase in selenium in the spleen of mice treated with HA-HDAPPs delivered via injection. Error bars represent standard error of the mean.
